# Plasma proteomic profiling of bacterial cold water disease-resistant and -susceptible rainbow trout lines and biomarker discovery

**DOI:** 10.3389/fimmu.2023.1265386

**Published:** 2023-10-20

**Authors:** Gregory D. Wiens, David P. Marancik, Christopher C. Chadwick, Keira Osbourn, Ross M. Reid, Timothy D. Leeds

**Affiliations:** ^1^ National Center for Cool and Cold Water Aquaculture, Agricultural Research Service, U.S. Department of Agriculture (USDA), Kearneysville, WV, United States; ^2^ Department of Pathobiology, School of Veterinary Medicine, St. George’s University, True Blue, Grenada; ^3^ Life Diagnostics, Inc. and Veterinary Biomarkers Inc., West Chester, PA, United States

**Keywords:** *Flavobacterium psychrophilum*, bacterial cold water disease, disease resistance, biomarker, complement C1q-like protein 3, complement factor H-like 1

## Abstract

Genetic variation for disease resistance is present in salmonid fish; however, the molecular basis is poorly understood, and biomarkers of disease susceptibility/resistance are unavailable. Previously, we selected a line of rainbow trout for high survival following standardized challenge with *Flavobacterium psychrophilum* (*Fp*), the causative agent of bacterial cold water disease. The resistant line (ARS-Fp-R) exhibits over 60 percentage points higher survival compared to a reference susceptible line (ARS-Fp-S). To gain insight into the differential host response between genetic lines, we compared the plasma proteomes from day 6 after intramuscular challenge. Pooled plasma from unhandled, PBS-injected, and *Fp*-injected groups were simultaneously analyzed using a TMT 6-plex label, and the relative abundance of 513 proteins was determined. Data are available via ProteomeXchange, with identifier PXD041308, and the relative protein abundance values were compared to mRNA measured from a prior, whole-body RNA-seq dataset. Our results identified a subset of differentially abundant intracellular proteins was identified, including troponin and myosin, which were not transcriptionally regulated, suggesting that these proteins were released into plasma following pathogen-induced tissue damage. A separate subset of high-abundance, secreted proteins were transcriptionally regulated in infected fish. The highest differentially expressed protein was a C1q family member (designated complement C1q-like protein 3; C1q-LP3) that was upregulated over 20-fold in the infected susceptible line while only modestly upregulated, 1.8-fold, in the infected resistant line. Validation of biomarkers was performed using immunoassays and C1q-LP3, skeletal muscle troponin C, cathelcidin 2, haptoglobin, leptin, and growth and differentiation factor 15 exhibited elevated concentration in susceptible line plasma. Complement factor H-like 1 exhibited higher abundance in the resistant line compared to the susceptible line in both control and challenged fish and thus was a baseline differentiator between lines. C1q-LP3 and STNC were elevated in Atlantic salmon plasma following experimental challenge with *Fp*. In summary, these findings further the understanding of the differential host response to *Fp* and identifies salmonid biomarkers that may have use for genetic line evaluation and on-farm health monitoring.

## 1 Introduction

The potential for selective breeding to improve fish health and welfare has long been recognized (1, 2), and within the past two decades, breeding programs have dedicated efforts towards understanding and increasing disease resistance in resource and farmed fish populations (reviewed in (3–8)). The high fecundity, external fertilization, and the temperature synchronization of early development allow evaluation of a portion of each full-sib family by standardized challenge, and then applying selection and propagation to unexposed, full-siblings. Post-challenge survival is the phenotype typically used for the selection decision as surrogate markers are generally unavailable (8) and large-effect disease resistance genes have been elusive, with some exceptions (9). Previously, we (10, 11) and others (12–15) have reported family-based evaluation and breeding of rainbow trout for increased innate resistance against *Flavobacterium psychrophilum* (*Fp*), the causative agent of bacterial cold water disease (BCWD). This pathogen causes considerable losses to the U.S. rainbow trout aquaculture industry and to salmonid populations worldwide (16–19). Infection of rainbow trout with *F. psychrophilum* typically results in mortality, ranging from 2% to 30% of the population on-farm, with higher mortality caused by coinfection with infectious hematopoietic necrosis virus (20, 21). Disease prevention is difficult as the pathogen is geographically widespread, fish are often affected at early life stage, and limited chemotherapeutants are available for treatment. There is currently no commercial vaccine available in the U.S., although killed, subunit, and live-attenuated vaccines are actively being evaluated, and several vaccines have demonstrated protection under laboratory and field conditions (22–24).

Multiple generations of selection have been applied to an odd-year spawning line of pedigreed rainbow trout, developed from the intercrossing of four domesticated founder strains (11), using an intraperitoneal injection-challenge model (25). The improved genetic line has been designated ARS-Fp-R, and a susceptible line, designated ARS-Fp-S, has been developed from the same resource population (26). Following standardized challenge, the ARS-Fp-R line exhibits higher survival, lower bacterial load, and greater naïve spleen size (25, 27, 28) while biochemical reference intervals of naïve fish do not differ between lines (29). After three generations of selection, the resistant line exhibited higher on-farm survival at locations experiencing natural outbreaks of BCWD within adjacent or co-mingled trout populations (26, 30).

Gene expression studies comparing infected resistant and susceptible fish have generally found a higher number of regulated genes in susceptible fish, correlated with higher pathogen load (31–38), although the opposite pattern has also been observed (39). Previously, we reported analysis of whole-body gene expression, using RNA-Seq, to quantify changes in gene transcript abundance between genetic lines and identified 1,884 genes (4.0% of the protein coding transcripts identified in the rainbow trout genome) that exhibited differential transcript expression between infected and mock-challenged genetic lines (27). The ARS-Fp-S line fish exhibited a greater number of regulated genes, and expression levels were positively correlated with *Fp* load (27). This pattern is also observed between clonal lines of resistant and susceptible rainbow trout (31, 40). Regulated genes included interleukins, tumor necrosis factor (TNF) receptor superfamily members, chemokines, complement components, acute phase response genes, nod-like receptor family members, and genes putatively involved in metabolism and wound healing. *Flavobacterium psychrophilum* causes skeletal muscle cell apoptosis and modulates gene expression associated with caspase activity, ubiquitin proteasome system, and muscle atrophy (41, 42).

At present, limited information exists on the proteomic response to *Fp* infection (43). The goals of this study were threefold: (1) identify proteomic differences between resistant and susceptible lines of fish both at baseline and during infection; (2) compare this proteomic dataset to a previously published whole-body RNA-seq dataset (27); and (3) develop specific and rapid immunoassays to monitor biomarkers that differ between lines. Here, we compared the plasma proteomes on day 6 following intramuscular, injection challenge, a time point when differential mortality is typically observed. Pooled plasma from unhandled, PBS-injected, and *Fp*-injected groups was simultaneously analyzed using a TMT 6-plex labeling protocol. Several biomarkers were identified for in-depth study and validated in Atlantic salmon (*Salmo salar*) and a commercial population of rainbow trout challenged with *Fp*.

## 2 Methods

### 2.1 Experimental animals

The ARS-Fp-R and ARS-Fp-S genetic lines were derived from the same founder population developed in 2005, and thus differed because of artificial selection for post-challenge survival. While applying selection pressure for improved survival, genetic diversity was maintained, and cumulative pedigree-based estimates of inbreeding were less than 8% per line. Rainbow trout broodstock from each line are spawned during a 5-week period in February and March at the NCCCWA. The fish were produced with single-sire × single-dam mating within genetic lines between naturally maturing 2-year-old females and 2-year-old neomales each having been subjected to 4 generations of selection (10, 26). Eggs were incubated separately by full-sib family in upwelling jars, and water temperature in the incubation jars was manipulated so that all families would hatch within a 1-week period (10). After hatching, fish were reared in flow-through spring water (approximately 12.5°C), and fed daily a commercial diet (Zeigler Bros., Inc., Gardners, PA) from swim-up through the last experimental day. All broodstock were certified to be free of common salmonid bacterial and viral pathogens by independent diagnostic laboratories as described previously (10, 26).

### 2.2 Bacterial challenge isolate

*Flavobacterium psychrophilum* CSF259-93 was previously isolated from a BCWD field case and belongs to MLST sequence type 10 (44), multiplex PCR type II (45), and O-polysaccharide composed of L-Rhamnose, 2-acetamido-2-deoxy-L-fucose, and 2-acetamido-4-R1-2,4-dideoxy-D-quinovose (46, 47). Stock culture was maintained at −80°C in TYES media supplemented with 10% (v/v) glycerol. This bacterial isolate has been consistently utilized as the challenge strain within the selective breeding program (48). Frozen stock was cultivated on TYES media for 5 days at 15°C prior to experimental infection.

### 2.3 Design of proteomics experiment and challenge

The ARS-Fp-R and ARS-Fp-S lines were each represented by 10 pedigreed families. At the time of the experiment, average fish weight was 396 ± 37 g, and age was 341 days post-hatch. Fish were PIT tagged (Avid Identification Systems Inc., Norco, CA and Biomark, Inc., Boise, ID) for identification 2 months prior to the experiment and four ARS-Fp-R and four ARS-Fp-S line groups (*n* = 10 fish/group) were generated that contained one fish from each family. Each group was randomly split between two 50-L tanks receiving 2 L min^−1^ of 13°C flow-through spring water for a total of 16 tanks and acclimated for 1 week.

**Figure 1:**
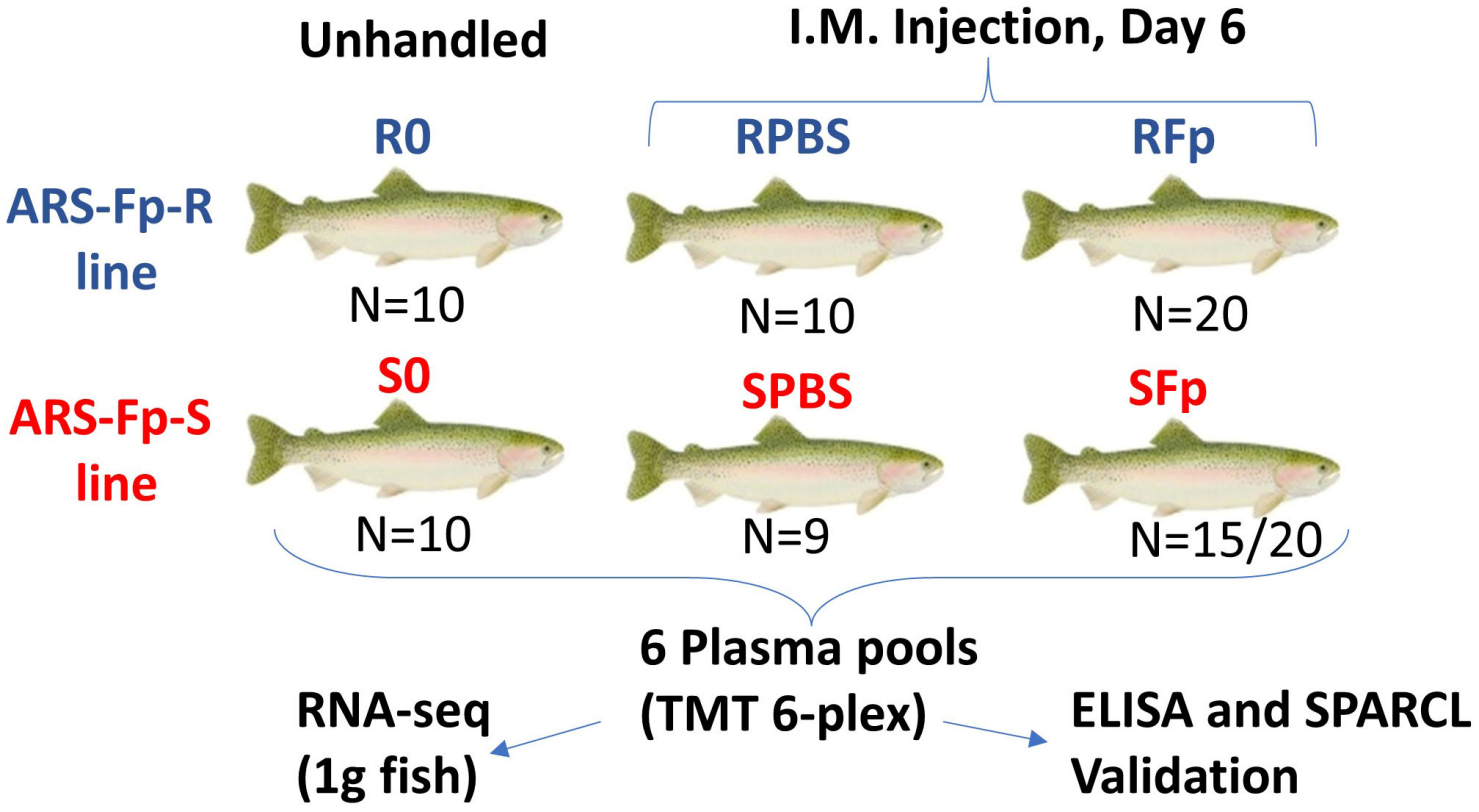
Experimental design comparing plasma proteomes between genetic lines and infection status. Six groups were compared in this study and are shown by genetic line and infection status. N is the number of fish in each group contributing to the plasma pool. Proteomic changes measured by TMT 6-plex were compared to a published RNA-seq dataset from 1 g fish, and candidate biomarkers were validated by ELISA and/or SPARCL immunoassays. In the SFp group, 15 fish out of 20 initial study fish contributed to the pool.

On day 0, one ARS-Fp-R and one ARS-Fp-S line group was sampled to establish unhandled or resting plasma protein levels (**Figure 1**). Fish were euthanized with 250 mg/L tricaine methanesulfonate (Tricaine-S, Western Chemical, Inc., Ferndale, WA) and approximately 1 mL of blood was collected from the caudal vein using a 3-mL syringe and a 25-G 1/2 inch needle (Kendall Monoject, Mansfield, MA) and placed into 3-mL lithium heparin tubes (Greiner Bio-One, Monroe, NC). Collection tubes were centrifuged within 20 min of collection at 1,000 × *g* for 15 min at 15°C. Plasma was collected into 1.5 mL microcentrifuge tubes and frozen at −80°C.

The remaining fish were anesthetized with tricaine methanesulfonate (100 mg/L) and two groups (*n* = 20 in four tanks) from each genetic line were challenged intramuscularly within the left epaxial muscle with 1.55 × 10^8^ cfu of *F. psychrophilum* CSF 259-93 in 200 µL of chilled PBS using a repeater pipette (Eppendorf, Hauppauge, NY) fitted with a 22-G 1-inch needle. One ARS-Fp-R and ARS-Fp-S line group was challenged with 200 µL of chilled PBS. Fish were euthanized on day 6 post-infection and 1 mL of blood was collected and processed as described above. Packed cell volume was measured as previously described (29). Splenic samples were aseptically collected for *F. psychrophilum*-specific qPCR to determine pathogen load, as previously described (49). qPCR samples were tested using two technical replicates and values were averaged. Four ARS-Fp-S line fish died prior to the day 6 post-infection sample day and one PBS-challenged ARS-Fp-S line fish died for reasons unrelated to the challenge and one plasma sample from the SFp group was lost during the collection process. The final sample size for proteomic analyses was *n* = 15 in the ARS-Fp-S line and *n* = 20 in the ARS-Fp-R line for *F. psychrophilum*-challenged fish and *n* = 9 in the ARS-Fp-S line and *n* = 10 in the ARS-Fp-R line for PBS-challenged fish (**Supplementary Data 1**).

To evaluate whether several of the biomarkers identified in this study might be informative in other rainbow trout stocks and salmonid species, Atlantic salmon (StofnFiskur stock, Benchmark Genetics) were maintained in freshwater in 965-L tanks (*n* = 10 fish/tank) and were challenged at 209 days post-hatch (590 ± 88g). A commercial population of May spawning rainbow trout (Troutlodge, WA) were held in 12-L tanks (*n* = 20 fish/tank) and were challenged at 127 days post-hatch (14.2 ± 1.0 g). Triplicate tanks were used to assess mortality and duplicate tanks were used to sample plasma post-challenge.

### 2.4 TMT 6-plex and LC-MS/MS analysis

Plasma protein levels were measured in each pool by spectrophotometry (Nanodrop ND-1000, Wilmington, DE) to confirm sufficient protein concentrations. Pools of plasma were made for each group of fish using 50 µL of plasma/fish and pooled samples were sent on dry ice to Cornell University Proteomics and Mass Spectrometry Core Facility (Ithaca, NY) for proteomic analyses. Pooled sample protein levels were quantified by A_280_ absorbance using a Nanodrop and SDS-PAGE and the values agreed (**Supplementary Figure 1**).

Protein expression differences between each of the six groups was quantified by isobaric tags for relative quantitation (TMT 6-plex) profiling. One-hundred-microliter aliquots of each of the samples were labeled with TMT 6-plex tags as follows: ARS-Fp-R day 0 (R0) = 126, ARS-Fp-S Day 0 (S0) = 127, ARS-Fp-R PBS challenged (RPBS) = 128, ARS-Fp-S PBS challenged (SPBS) = 129, ARS-Fp-R *Fp* challenged (RFp) = 130, and ARS-Fp-S *Fp* challenged (SFp) = 131. The mixed tag labeled samples were constructed by first-dimension high pH RP separation of tryptic peptide mixtures by Ultimate3000 MDLC platform with built-in fraction collection option, autosampler, and UV detection (Dionex, Sunnyvale, CA). The tandem mass tagged tryptic peptides were reconstituted in 20 mM ammonium formate (NH_4_FA) pH 9.5 in water (buffer A) and loaded onto an XTerra^®^ MS C18 column (3.5 µm, 2.1 × 150 mm, in water) (Waters Corp, Milford, MA) with buffer A and 80% acetonitrile (ACN)/20% 20 mM NH_4_FA (buffer B).

Liquid chromatography was performed using a gradient from 10% to 45% of buffer B for 30 min at a flow rate of 200 µL/min. Forty-eight fractions were collected at 1-min intervals in a 96-well plate and pooled into a total of 10 fractions based on UV absorbance at 214 nm. Fractions were pooled into the final 10 fractions by disparate first-dimension fractions (retention time multiplexing) using concatenation strategy. All 10 pooled peptide fractions were dried and reconstituted in 2% ACN/0.5% formic acid for Nano LC-MS/MS analysis.

2D-LC-MS/MS analysis was performed on equal mixtures of tag-labeled digests. Nano LC-MS/MS analysis was carried out using an LTQ-Orbitrap Velos mass spectrometer (Thermo-Fisher Scientific, San Jose, CA) equipped with nano ion source via high-energy collision dissociation (HCD) and interfaced with an UltiMate3000 RSLC nano system (Dionex, Sunnyvale, CA). Ten-milliliter aliquots of each pH RP peptide fraction were injected onto a PepMap C18 trap column (5 µm, 300 µm × 5 mm) for desalting at a 20 mL/min flow rate. Fractions were then separated on a PepMap C-18 RP nano column (3 µm, 75 µm × 15 cm) and eluted for 90 min in a gradient of 5% to 38% ACN in 0.1% formic acid at 300 nL/min followed by a 3-min ramping to 95% ACN–0.1% FA and a 5-min holding at 95% ACN–0.1% FA. The column was re-equilibrated with 2% ACN–0.1% FA for 20 min prior to the next run.

The eluted peptides were detected by Orbitrap through nano ion source containing a 10-µm analyte emitter (New Objective, Woburn, MA). The Orbitrap Velos was operated in positive ion mode with nano spray voltage set at 1.5 kV and a source temperature at 275°C with nitrogen as the collision gas. Calibration was performed internally using the background ion signal at m/z 445.120025 as a lock mass or externally using a Fourier transform (FT) mass analyzer. The instrument was run on data-dependent acquisition (DDA) mode using FT mass analyzer for survey MS scans of precursor ions followed by 10 data-dependent HCD-MS/MS scans for precursor peptides with multiple charged ions above a threshold ion count of 7,500 with a normalized collision energy of 45%. MS survey scans were conducted at a resolution of 30,000 FWHM at m/z 400 for the mass range of m/z 400–1,400 and MS/MS scans were conducted at 7,500 resolution for the mass range of m/z 100–2,000. All data were acquired under Xcalibur 2.1 operation software (Thermo-Fisher Scientific, San Jose, CA). All MS and MS/MS raw spectra data from TMT 6-plex experiments were processed using Proteome Discoverer 2.3 (PD2.3, Thermo Scientific, San Jose, CA). Normalization was accomplished using the Total Peptide Amount setting in the Normalization Mode Parameter of Proteome Discoverer 2.3.

The MS/MS spectra were searched against *Oncorhynchus mykiss* database sequence accession GCF_002163495.1_Omyk1.0_protein.faa (50) with 62,608 separate sequences using the described workflow (**Supplementary Data 2**_Omyk1.0 Analysis Parameters). Oxidation of M and deamidation of N and Q were specified as dynamic modifications of amino acid residues; protein N-terminal acetylation was set as a variable modification; TMT-6plex of K and carbamidomethyl C were specified as a static modification. Proteins with at least one unique peptide were identified and abundance was compared (*n* = 513, **Supplementary Data 2**_ Omyk1.0 Protein Ratios). Proteins with only one peptide (*n* = 98) within this dataset should be considered as a tentative identification. During the analysis of the dataset, an updated rainbow trout genome (USDA_OmykA_1.1, GCF_013265735.2) became available (51). MS/MS spectra were searched against the updated rainbow trout genome as well as *F. psychrophilum* CSF59-93 genome, GCA_000739395.1, using parameters as previously described (**Supplementary Data 2**_Analysis Parameters, OmykA_1.1). The mass spectrometry proteomics data have been deposited to the ProteomeXchange Consortium via the PRIDE (52) partner repository with the dataset identifier PXD041308.

### 2.5 Identification of differentially expressed plasma proteins

Differences in protein abundance were determined between groups by calculating the ratios between the peak areas of the TMT 6-plex reporter groups. Ratios were further sorted by proteins that exhibited a ≥1.2-fold change. Owing to the large number of proteins, a ≥2-fold change was used as a benchmark to compare protein levels of *Fp*-challenged and PBS-challenged groups to day 0 groups. Hierarchical clustering of both samples and variables was performed using Qlucore Omics Explorer 3.0 (Lund, Sweden).

### 2.6 Comparison of proteomic and transcriptomic response to infection

Proteins found to be differentially expressed between *Fp*-challenged ARS-Fp-R and ARS-Fp-S line fish were compared with a previously performed RNA-seq analysis that characterized whole-body gene transcript abundance between the same infected ARS-Fp-R and ARS-Fp-S lines on day 5 post-infection (27). These transcriptomic data from the resistant and susceptible lines were reanalyzed using the same rainbow trout reference genome (accession GCF_002163495.1_Omyk1.0) allowing deductions to be made about corresponding protein and gene expression differences between genetic lines. NCBI protein IDs were matched to gene IDs within the transcriptomic dataset (*n* = 507) and linear regression was used to assess the fold-difference similarity within genetic lines (GraphPad Prism Version 5.0, La Jolla, CA) (*p* < 0.05).

GO pathway enrichment analyses were performed after converting ≥2-fold change. regulated NCBI protein/gene ID’s into Ensembl (v204) gene IDs and analyzed using g:Profiler (53). Gene list was input as an ordered query, analyzed against all known genes, and corrected for multiple comparisons using the g:SCS algorithm.

### 2.7 ELISA and SPARCL™ assays

Enzyme-linked immunosorbent assay (ELISA) and Spatial proximity analyte reagent capture luminescence (SPARCL™) (54) assay were run according to manufacturer’s directions. ELISAs (Life Diagnostics Inc.) were used to measure complement C1q-like protein 3 (C1q-LP3), complement factor H-like protein (CFHL-1), haptoglobin (Hp), cathelicidin 2 (Cath2), growth and differentiation factor 15 (GDF-15), and leptin (Lept). SPARCL™ assays (Veterinary Biomarkers Inc.) were used to measure C1q-LP3, cardiac/slow-twitch skeletal muscle troponin C (CTNC), fast-twitch skeletal muscle troponin C (STNC), and haptoglobin. All assays except the CTNC and STNC SPARCL™ assays utilize affinity-purified polyclonal rabbit antibodies generated against either rainbow trout or Atlantic salmon recombinant proteins expressed and purified from *Escherichia coli*. The CTNC and STNC SPARCL™ assays use antibodies generated against native human CTNC and Atlantic salmon STNC. Proteins used to generate the respective antibodies were used as standards in the assays. To measure biomarker concentration in plasma, the dilution was adjusted to fall within the range of each assay’s standard curve. ELISA dilution range for each assay was as follows: C1q-LP3 assay, 100- to 600-fold; CFHL-1 assay, 800- to 6,400-fold; Hp assay, 100- to 500-fold; Cath2 assay, 200- to 1,000-fold; GDF-15 and Lept assays, 4-fold. Samples were tested repeatedly in two or more independent assays performed at separate physical locations. Parallelism was demonstrated for all assays except the leptin ELISA, for which an average CV of 21% was obtained when testing six samples at dilutions ranging from 4- to 64-fold; all samples were therefore tested at a dilution of 1:4.

### 2.8 Protein modeling, domain analysis, and phylogeny

Homology modeling of C1q-LP3 was performed using Swiss-Model and used to estimate stoichiometry (55). Domain analyses were performed using SMART (56) and PROSITE (57). The evolutionary history of rainbow trout CFHL-1 was inferred by using the Maximum Likelihood method and JTT matrix-based model (58) using MEGA11 (59). The bootstrap consensus tree was inferred from 1,000 replicates. Initial tree(s) for the heuristic search were obtained automatically by applying Neighbor-Join and BioNJ algorithms to a matrix of pairwise distances estimated using the JTT model, and then selecting the topology with superior log likelihood value. There were a total of 1,868 positions in the final dataset.

### 2.9 Statistical analyses

Statistical comparison of bacterial loads between lines was performed by one-way ANOVA using GraphPad Prism v9.0 (La Jolla, CA). Quantitative PCR bacterial load data were log10-transformed prior to statistical analyses. Two ARS-Fp-R line fish had mean values below the average qPCR limit of quantification (0.5 GE/100 ng DNA, C_t_>36.8) and were not included in the load comparison. All statistics were run with a significance level of *p* < 0.05.

Biomarker values were compared by ANOVA using a Kruskal–Wallis test with Dunn’s multiple comparisons calculated using GraphPad Prism v9.0. Linear regression, 95% confidence intervals, and Spearman correlations were calculated using GraphPad Prism v9.0. Principal component analysis, heat maps, hierarchal clustering, and network analyses were performed using Qlucore Omics Explorer v3.8 (Qlucore, Lund Sweden).

## 3 Results

### 3.1 Genetic lines differ in mortality, Fp load, and packed cell volume post-challenge

**Figure 2:**
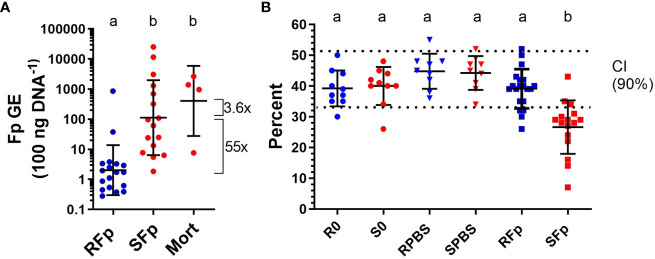
Bacterial load and plasma packed cell volume on day 6 post-infection. **(A)** *Flavobacterium psychrophilum* abundance in spleen tissue and expressed as genomic equivalents per 100 ng of input sample DNA (geometric mean ± sd). **(B)** Whole blood packed cell volume (mean ± sd). The 90% confidence intervals for healthy fish are indicated by dotted lines (from Marancik et al. (29)). One-way ANOVA with letters indicating significant difference (*p* < 0.05).

By day 6 post-challenge, 20% (*n* = 4/20) of the susceptible line fish had died from infection while all the challenged, resistant line fish were alive (*n* = 20) at the time of sampling. The geometric mean of splenic bacterial load in infected ARS-Fp-R line fish (2.03 GE 100 ng DNA^−1^, *n* = 18) was 55-fold lower than the mean load in ARS-Fp-S line fish (112.6 GE 100 ng DNA^−1^, *n* = 16, *p* < 0.001, one-way ANOVA, **Figure 2A**). The load in the four susceptible line fish that died was 3.6-fold higher that the live-sampled, susceptible line fish. Analysis of load by family, identified three ARS-Fp-S line families (473, 335, and 388) with lower relative loads and one ARS-Fp-R family (476) with higher load indicating some family-based variation within each genetic line (**Supplementary Figure 2A**). However, these families did not differ from respective mean line values when phenotyped for survival at 80 days post-hatch (**Supplementary Figure 2B**) or by breeding values estimated using a linear animal model (10) (**Supplementary Figure 2C**). The mean packed cell volume of live-sampled ARS-Fp-S line fish was 26.6% and significantly lower than ARS-Fp-R line fish, 39.1%, (*p* < 0.001, one-way ANOVA). The packed cell volume of ARS-Fp-R line fish did not differ from the PBS-injected or unhandled fish (**Figure 2B**). Analysis of packed cell volume by family identified most ARS-Fp-S line values fell outside the reference interval (**Supplementary Figure 2D**).

### 3.2 Differentially abundant proteins between groups

The 2D-LC-MS/MS analysis identified 38,763 peptide spectra matched for proteins that corresponded to 528 protein accessions from the Swanson clonal rainbow trout line reference genome (Omyk 1.0) with an estimated false discovery rate of 0.05. Abundance ratios for each of the groups could be calculated for 513 proteins (**Supplementary Data 2**, see Omyk_1.0 Protein Ratios). No significant matches to *F. psychrophilum* CSF259-93 proteins were detected within the dataset.

Pairwise comparisons between groups, using both relaxed and stringent cutoff criteria (1.2-fold and 2-fold change, respectively) were used to identify proteins with divergent abundances due to infection, genetic line, and injection/handling. Depending on cutoff criteria and group comparison, between 3 and 283 proteins were identified as differentially abundant (**Table 1**). The 1.2-fold criteria have been used in prior studies to identify differentially regulated proteins (60, 61); however, utilization of the more stringent criteria (2-fold) reduced the number of proteins regulated by injection/handling procedure (**Table 1**). The number of regulated proteins in the RPBS/RO comparison was reduced from 90 to 3, and in the SPBS/SO comparison, the number was reduced from 70 to 5. Subsequent downstream analyses focused on the proteins belonging to the ≥2-fold criteria.

**Table 1:** Number of proteins with altered relative abundance between genetic line, infection, and handling.

	≥1.2-f.c.^a^			≥2-f.c.^b^		
Group Comparison	Total	Up	Down	Total	Up	Down
Infection						
SFp/SPBS	272	146	126	61	47	14
SFp/S0	283	140	143	61	50	11
RFp/RPBS	241	111	130	35	23	12
RFp/R0	246	116	130	31	23	8
Genetic Line						
SFp/RFp	209	127	82	40	33	7
SPBS/RPBS	149	70	79	21	7	14
S0/R0	151	73	78	14	7	7
Injection						
RPBS/R0	90	43	47	3	1	2
SPBS/S0	70	32	38	5	3	2

Fold-change comparisons were calculated between day 0 (D0), PBS-challenged (PBS), and *Flavobacterium psychrophilum* challenged (Fp) by genetic line. Differential protein abundance was defined as either ≥1.2-fold change (f.c.) or ≥2-f.c.

a Up = Group 1/Group 2 ≥ 1.2; Down = Group 1/Group 2 ≤ 0.83.

b Up = Group 1/Group 2 ≥ 2.0; Down = Group 1/Group 2 ≤ 0.5.

### 3.3 Infection regulated proteins

**Figure 3:**
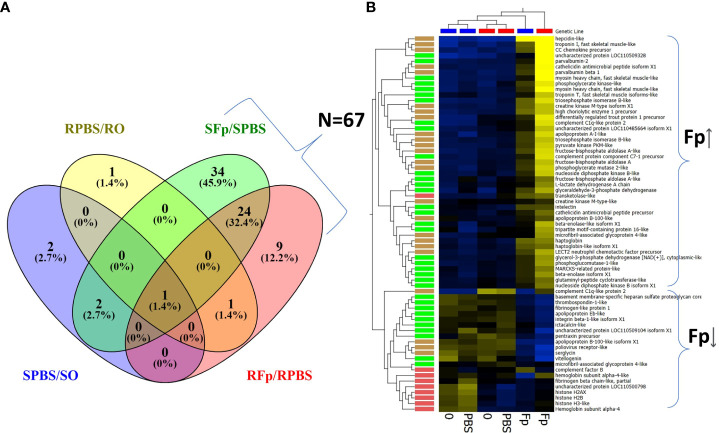
Infection-regulated proteins with ≥2-fold change. **(A)** Venn diagram of proteins either shared or unique to each group: SFp/SPBS (*n* = 61), RFp/RPBS (*n* = 35), SPBS/S0 (*n* = 5), RPBS/R0 (*n* = 3). **(B)** Heat map of infection-regulated proteins (*n* = 67) specific to either RFp/RPBS or SFp/SPBS groups determined from the fold-change comparisons shown in **(A)**. Brackets indicate broad infection regulated categories of either increased abundance (Fp ↑) or decreased abundance (Fp↓). Hierarchal clustering of both samples and proteins identifies patterns of expression and color coding indicate the following groups: unique to SFp/PBS, green; unique to RFp/RPBS, red; shared between groups, brown. Heat map: yellow increased, blue decreased fold change.

Overall, there was a greater number of infection-regulated proteins in the infected susceptible line fish as compared to the resistant line fish, SFp/SPBS *n* = 61 vs. RFp/RPBS *n* = 35, respectively, and the same trend was also observed when comparing infected relative to unhandled fish (SFp/S0 *n* = 61 vs. RFp/R0 *n* = 31). To identify infection-specific proteins, the SPBS/S0 and RPBS/R0 differences were excluded, and a total of 67 proteins remained (**Figure 3A**). Thirty-four proteins were unique to the SFp/SPBS group and 9 were unique to the RFp/RPBS group, while 24 were identified in both groups. Patterns of expression were examined by hierarchal clustering of normalized abundance values (**Figure 3B**) with most proteins, *n* = 46, increasing in abundance due to infection.

The most highly upregulated protein in the susceptible line was an uncharacterized protein, XP_021445926.1, a product of gene locus LOC110509328. This protein was over 20-fold more abundant in the infected ARS-Fp-S line in comparison to either the PBS-injected fish or unhandled susceptible line fish (**Table 2**). Characterization of this protein, complement C1q-like protein 3, is described in further detail in *section 3.6*. Other highly upregulated proteins included hepcidin-like (XP_021416707.1), serum amyloid A-5 protein-like (XP_021463248.1), CC chemokine (NP_001117839.1), and cathelicidin (XP_021466896.1). Muscle proteins were also differentially abundant including troponin I (XP_021454537.1), myosin heavy chain (XP_021452021.1), and parvalbumin beta 1 and 2 (XP_021414352.1, NP_001182340.1) (**Table 2**). The most highly infection-regulated proteins in the resistant line were hepcidin, CC chemokine, SAA, troponin, and haptoglobin (XP_021441697.1) (**Table 2**). These proteins exhibited lower fold change values in the resistant line as compared to the susceptible line.

**Table 2:** Top infection-upregulated proteins by genetic line.

Accession	Description	# Peptides	Ratio Fp/PBS	Ratio Fp/S0
Susceptible line (ARS-Fp-S)				
XP_021445926.1	LOC110509328 (**C1q-LP3**)	6	21.3	27.6
XP_021416707.1	Hepcidin-like	2	15.2	16.0
XP_021463248.1	Serum amyloid A-5 protein-like	1	11.9	5.8
XP_021454537.1	Troponin I, fast skeletal muscle-like	2	10.9	13.3
NP_001117839.1	CC chemokine precursor	1	9.9	8.7
XP_021452021.1	Myosin heavy chain, fast skeletal muscle-like	25	7.8	8.5
XP_021414352.1	Parvalbumin beta 1	7	7.1	7.8
NP_001182340.1	Parvalbumin-2	5	6.4	6.1
XP_021466896.1	Cathelicidin antimicrobial peptide isoform X1	3	5.8	5.9
XP_021431248.1	Myosin heavy chain, fast skeletal muscle-like	4	5.2	6.1
Resistant line (ARS-Fp-R)				
XP_021416707.1	Hepcidin-like	2	8.0	9.5
NP_001117839.1	CC chemokine precursor	1	3.1	2.8
XP_021463248.1	Serum amyloid A-5 protein-like	1	3.1	1.2
XP_021462825.1	Haptoglobin	12	2.9	2.2
XP_021454537.1	Troponin I, fast skeletal muscle-like	2	2.8	3.9
NP_001158583.1	High choriolytic enzyme 1 precursor	3	2.7	3.0
XP_021417729.1	Triosephosphate isomerase B-like	12	2.7	2.8
XP_021453338.1	Apolipoprotein A-I-like	14	2.7	2.0
XP_021473429.1	Creatine kinase M-type isoform X1	14	2.4	2.6
XP_021441697.1	Haptoglobin-like isoform X1	12	2.3	1.9

Proteins downregulated by infection included serglycin (XP_021436653.1), poliovirus receptor-like (XP_021427658.1), LOC110509104 (XP_021445743.1), and apolipoprotein B-100-like (XP_021467197.1 and XP_021467201.1) (**Table 3**). Multiple histone proteins (XP_021424008.1, XP_021474674.1, XP_021448780.1, and XP_021424008.1) were lower in infected resistant line fish (**Table 3**).

**Table 3:** Top infection-downregulated proteins by genetic line.

Accession	Description	# Peptides	Ratio Fp/PBS	Ratio Fp/S0
Susceptible line (ARS-Fp-S)				
XP_021427658.1	Poliovirus receptor-like	1	0.25	0.25
XP_021467197.1	Apolipoprotein B-100-like isoform X1	77	0.33	0.35
XP_021436653.1	Serglycin	1	0.34	0.30
XP_021445743.1	Uncharacterized protein LOC110509104	3	0.34	0.38
XP_021455471.1	Vitellogenin	5	0.36	0.23
XP_021467201.1	Apolipoprotein B-100-like	137	0.38	0.40
NP_001118193.1	Pentraxin precursor	4	0.41	0.35
XP_021451996.1	Apolipoprotein Eb-like	8	0.42	0.48
XP_021456715.1	Thrombospondin-1-like	2	0.43	0.41
XP_021476279.1	Integrin beta-1-like isoform X1	15	0.44	0.46
Resistant line (ARS-Fp-R)				
XP_021424008.1	Histone H1-like isoform X1	2	0.31	0.86
XP_021434006.1	Uncharacterized protein LOC110500798	1	0.37	0.52
XP_021413550.1	Hemoglobin subunit alpha-4-like	6	0.39	0.43
XP_021474674.1	Histone H2AX	1	0.42	0.52
XP_021448780.1	Histone H2B	3	0.45	0.54
XP_021467197.1	Apolipoprotein B-100-like isoform X1	77	0.46	0.41
XP_021477886.1	Fibrinogen beta chain-like, partial	3	0.48	0.78
XP_021436653.1	Serglycin	1	0.48	0.35
NP_001154036.1	Hemoglobin subunit alpha-4	5	0.49	0.52
XP_021424008.1	Histone H1-like isoform X1	2	0.49	0.61

### 3.4 Differentially abundant proteins between genetic lines

To identify proteins differentially regulated between genetic lines, proteins were sorted by infection (SFp/RFp ratio) and compared to PBS-injected fish (SPBS/RPBS) to determine if relative abundance initially differed under baseline conditions (**Table 4**). The uncharacterized protein product from LOC110509328 (XP_021445926.1) was highly induced in the susceptible line-infected fish relative to the resistant line-infected fish but did not differ between genetic lines in PBS-injected fish. Furthermore, it was induced only 1.8-fold in the RFp/RPBS comparison (**Supplementary Data 2**, see Omyk_1.0 Protein Ratios). Elevated fibrinogen alpha chain-like (XP_021477882.1) was initially identified as higher in both susceptible infected and susceptible PBS-injected fish, suggesting baseline difference in expression. However, when the dataset was reanalyzed using the OmykA1.1 reference, this protein was not differentially expressed (**Supplementary Data 2**, OmykA_1.1 Protein ratios). Three proteins were more highly expressed (twofold) in resistant line fish as compared to susceptible line fish (resulting in a low SFp/RFp ratio) including complement factor H-like (XP_021460375.1), trace amine-associated receptor 8a-like (XP_021435707.1), and ladder lectin-like (XP_021452379.1) (**Table 4**).

**Table 4:** Top protein differences between genetic lines.

Accession	Description	# Peptides	Ratio SFp/RFp	Ratio SPBS/RPBS
Higher in Susceptible line (ARS-Fp-S)				
XP_021445926.1	LOC110509328 (**C1q-LP3**)	6	16.0	1.3
XP_021477882.1	Fibrinogen alpha chain-like, partial^1^	19	11.8	9.9
XP_021452021.1	Myosin heavy chain, fast skeletal muscle-like	25	4.8	1.0
XP_021454537.1	Troponin I, fast skeletal muscle-like	2	4.0	1.0
NP_001182340.1	Parvalbumin-2	5	3.7	1.0
NP_001117839.1	CC chemokine precursor	1	3.5	1.1
XP_021413550.1	Hemoglobin subunit alpha-4-like	6	3.3	0.9
XP_021414352.1	Parvalbumin beta 1	7	3.3	1.0
XP_021431248.1	Myosin heavy chain, fast skeletal muscle-like	4	3.1	1.0
XP_021466896.1	Cathelicidin antimicrobial peptide isoform X1	3	2.9	1.0
Higher in Resistant line (ARS-Fp-R)				
XP_021460375.1	Complement factor H-like (**CFHL-1**)	15	0.26	0.25
XP_021435707.1	Trace amine-associated receptor 8a-like	1	0.28	0.25
XP_021452379.1	Ladder lectin-like^2^	7	0.32	0.26
XP_021445743.1	Uncharacterized protein LOC110509104	3	0.48	0.74
XP_021455471.1	Vitellogenin	5	0.49	0.86
XP_021449847.1	Type-4 ice-structuring protein LS-12-like	8	0.50	0.89
NP_001118067.1	Complement factor B	27	0.50	1.30
XP_021480137.1	Heme-binding protein 2-like	6	0.55	0.75
XP_021439053.1	C4b-binding protein alpha chain-like	12	0.56	0.53
XP_021427658.1	Poliovirus receptor-like	1	0.56	1.09

^1^Not differentially abundant using the OmykA_1.1 as a reference.

^2^Gene/protein absent in the OmykA_1.1 reference.

### 3.5 Correlation of plasma protein abundance and whole-body gene expression

**Figure 4:**
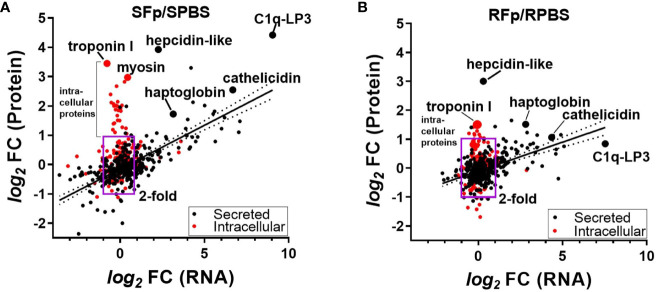
Comparison of the fold-change (f.c.) in protein (*y*-axis) and corresponding RNA (*x*-axis). **(A)** SFp/SPBS fold-change, *R* ^2 =^ 0.488, *p* < 0.0001 and **(B)** RFp/RPBS fold-change, *R* ^2 =^ 0.261, *p* < 0.0001. Box region marks twofold change in protein and RNA, and red colored circles indicate proteins that lack computationally predicted leader peptide and are thus likely intracellular proteins. Linear regression and 95% CI. Each slope is significantly non-zero. Individual proteins are labeled and are indicted by larger-sized circles.

To investigate the contribution of transcriptional regulation to differential protein abundance, we compared the proteomics dataset to a pooled-sample RNA-seq dataset from the same genetic lines sampled on day 5 post-challenge. There was a significant relationship (*p* < 0.0001) between protein and transcript abundance in both lines that were either PBS-injected or *Fp*-challenged (**Supplementary Figure 3**). This relationship between protein and transcript values was more clearly observed when the fold change was compared between groups (**Figure 4**). The *R* ^2^ value of the *log_2_* fc of the SFp/SPBS protein vs. RNA was 0.488, *p* < 0.0001 (**Figure 4A**), and for the RFp/RPBS group, *R* ^2 =^ 0.261 and *p* < 0.0001 (**Figure 4B**). In the susceptible line, a subset of proteins exhibited high fold change but were not transcriptionally regulated. Further inspection of these proteins indicated that they lacked signal peptide sequences and thus were likely intracellular (**Figure 4A**, **Supplementary Data 3**). GO annotation of the differentially expressed proteins identified cytoskeleton, troponin complex, and striated muscle in the cellular compartment enrichment (**Supplementary Figure 4**). The absence of transcriptional regulation and the elevated abundance in infected fish plasma suggest that these proteins may be released following pathogen-induced tissue damage. Consistent with this possibility, the fold increase in the resistant line comparison was lower than the susceptible line (**Figure 4B**).

### 3.6 Validation of biomarkers associated with the susceptible line

**Figure 5:**
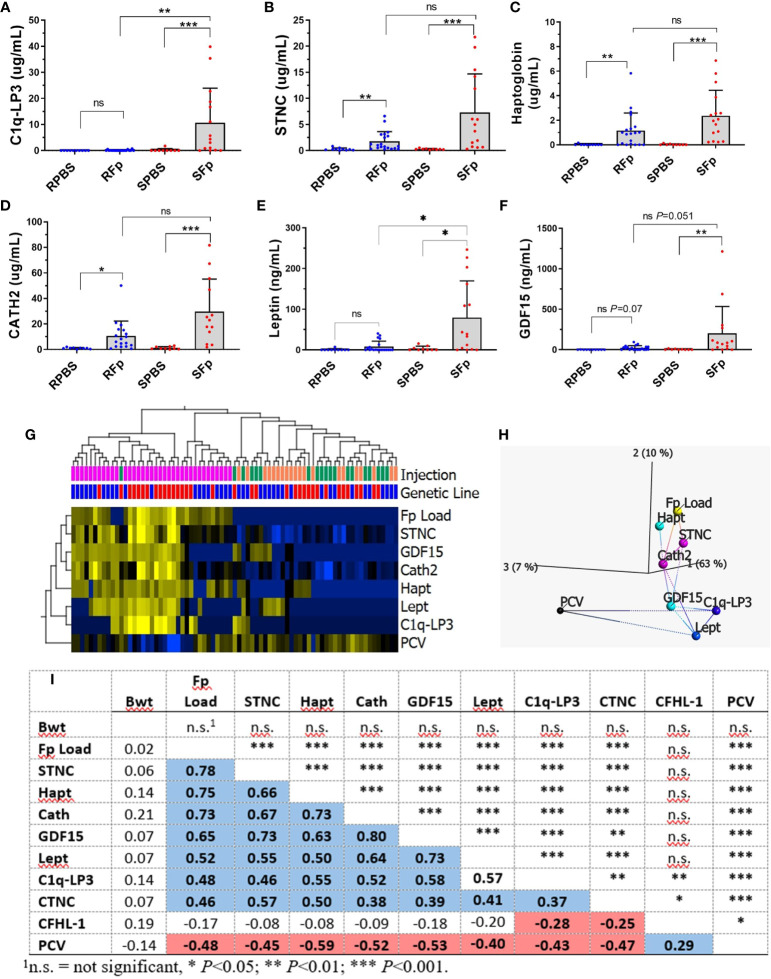
Validation of biomarkers (mean + sd). **(A)** C1q-LP3, **(B)** STNC, **(C)** Haptoglobin, **(D)** Cath2, **(E)** Leptin, and **(F)** GDF-15. Asterisks indicate significance values (* *p* < 0.05; ** *p* < 0.01; *** *p* < 0.001). **(G)** Heat map of normalized biomarker dataset markers significantly associated with infection (*q* < 0.05). **(H)** Nearest network analysis of variables and projection in top three principal component space. **(I)** Spearman correlations between each variable (bottom) and significance (top). Blue = significant positive correlation, red = significant negative correlation.

A subset of the proteins with elevated abundance in infected fish were chosen for further investigation and validation (**Figure 5**). Initial inspection of gene LOC110509328 (XP_021445926.1) indicated that the open reading frame was adjacent to a genomic gap and thus it was likely an incomplete protein prediction within the genome reference (Omyk_1.0). In an updated rainbow trout genome (OmykA_1.1), this gene model had no sequence gaps and encodes complement C1q-like protein 3, C1q-LP3 (**Supplementary Figure 5**; XP_036824181.1). The protein (256 aa) possesses a 19-aa leader peptide and the mature protein has an estimated Mw of 25.8 kDa. The C-terminus contains a C1q/TNFSF domain and is predicted to form a trimer (**Supplementary Figure 6A**). C1q-LP3 and haptoglobin ELISA and SPARCL™ assays were developed, which utilized either native or denaturing buffers, respectively, and assay results were highly correlated (*R* ^2 =^ 0.96), although the denaturing assay values were approximately 2.5-fold lower (**Supplementary Figures 7A–D**). Measurement of C1q-LP3 in plasma by native ELISA (**Figure 5A**) demonstrated a mean value of 10.7 μg/mL in infected susceptible line fish compared to either 0.20 μg/mL in SPBS-injected or 0.1 μg/mL in the S0 unhandled group, indicating specific elevation in infected susceptible line fish. Similar results were obtained using the SPARCL™ assay (data not shown). Immunoprecipitation of pooled RFp or SFp plasma identified a single ~26-kDa protein with higher abundance in the SFp plasma (**Supplementary Figure 6B**) consistent with ELISA results. The protein is present as a trimer as well as higher-order multimers when resolved using non-reducing SDS-PAGE (data not shown).

Skeletal muscle troponin C, haptoglobin, and cathelicidin 2 (Cath2) were significantly elevated in both infected resistant and susceptible line fish compared to PBS (**Figures 5B–D**), but significant differences between genetic lines were not detected within the PBS or Fp-injected groups. Mean plasma values in the SFp group were 7.3 μg/mL, 2.4 μg/mL, and 29.7 μg/mL, for STNC, haptoglobin, and Cath2, respectively. Additional assays were developed for genes that were highly transcriptionally regulated (27) but were not identified in the proteomics dataset. These included secreted growth and differentiation factor 15 (GDF-15) (XP_021444320.2) and leptin (NP_001139362.1). Mean plasma levels of GDF-15 and leptin in the SFp group were 201 ng/mL and 79 ng/mL, respectively. The mean levels in infected susceptible line were significantly greater than infected resistant line fish for leptin and approached significance for GDF-15 (**Figures 5E, F**). Levels of CNTC were elevated in the SFp group but did not significantly differ from the SPBS group (**Supplementary Figure 8**).

The relationship between biomarkers, bacterial load, and packed cell volume was analyzed with a combined analysis of the entire dataset (**Figure 5G**) and by genetic line (**Supplementary Figure 9**). In the combined analysis, variables significantly associated with infection were elevated STNC, GDF-15, Cath2, Hapt, Lept, and C1q-LP3 and decreased PCV. The relationship between each variable is shown by hierarchical clustering (**Figure 5G**), network analysis (**Figure 5H**), and Spearman correlation (**Figure 5I**). Splenic *Fp* load was most highly associated with STNC, Hapt, and Cath2 with a lower association with GDF-15, Lept, C1q-LP3, and CTNC (**Figure 5I**). Packed cell volume values were negatively correlated with each of the infection biomarkers, while there was no association between fish body weight and any variable. When analyzed by line, similar results were observed for the susceptible line (**Supplementary Figures 9A,C**) with the inclusion of CTNC as a biomarker. In the resistant line, only STNC, Hapt, GDF-15, and Cath2 were significant discriminators between the infected and control fish (**Supplementary Figures 9B, D**). Taken together, these data confirm overall trends observed in the proteomic analyses and establish both the shared and the unique response of each line.

### 3.7 Validation of a biomarker associated with the resistant line

**Figure 6:**
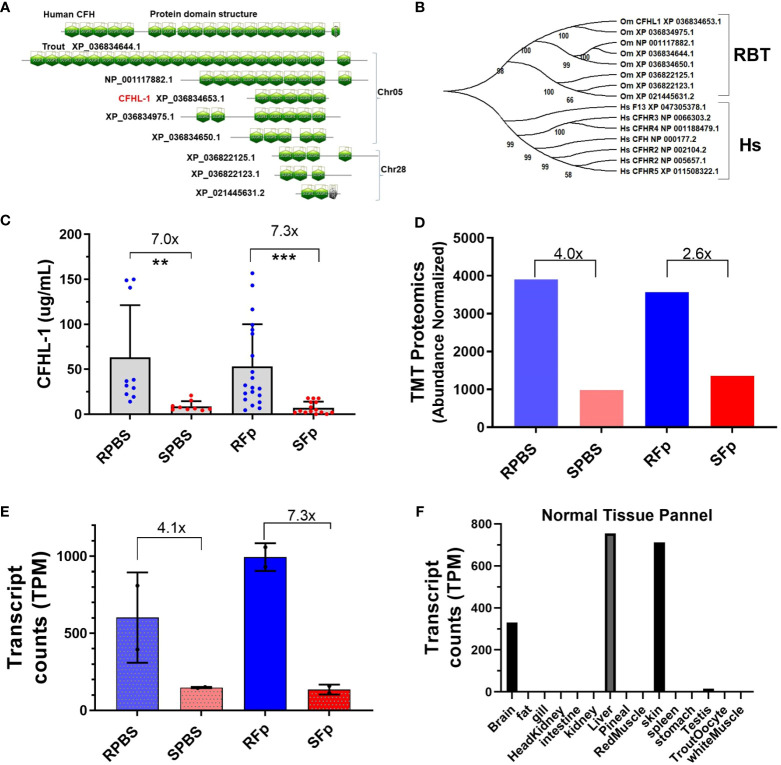
Complement factor H-like protein domains, phylogeny, and expression differences between lines. **(A)** SMART domain prediction of human CFH (NP_000177.2) and eight trout proteins based on gene models from the OmykA_1.1 assembly. **(B)** Phylogeny of human CFH and complement factor H-related proteins and trout proteins. **(C)**. Rainbow trout CHFL-1 ELISA. One-way ANOVA Kruskal–Wallis test with Dunn’s multiple comparisons test. Asterisks indicate significance values (** *p* < 0.01; *** *p* < 0.001). **(D)** TMT proteomics and relative abundance of trout CFHL-1 in plasma. **(E)** Day 5 *cfhl-1* gene expression values from Marancik et al. (27). **(F)** Transcript counts in normal tissue from Swanson clonal line.

While most induced proteins exhibited higher levels in susceptible line fish, several exhibited constitutively elevated abundance in resistant line fish (**Table 4**), which included complement factor H-like (XP_021460375.1). Genomic and domain analyses of the complement factor H-like protein identified eight sequence-related genes encoding proteins containing multiple sushi domains (**Figure 6A**). We designated the differentially abundant protein, CFHL-1, and this protein is located within a cluster with four additional genes on Chr 5 that includes a putative orthologue of human complement factor H, CFH. Three additional genes are located on Chr28. Rainbow trout complement factor H and the seven related proteins appear to be phylogenetically distinct from human complement factor H (CFH), complement factor H-related proteins (CFHR1-5), and coagulation factor XIIIB chain (F13B) that are clustered together on human Chr 1 (**Figure 6B**). The distinct evolutionary history of mammalian and fish proteins is consistent with a larger phylogenetic analysis of CFH orthologues available in Ensembl (data not shown). Rainbow trout CFHL-1 was sevenfold more abundant in both infected and PBS-injected resistant line fish plasma compared to susceptible line fish as measured by ELISA (**Figure 6C**). This is consistent with two- to fourfold elevation in the proteomics dataset (**Figure 6D**) and four- to sevenfold elevation in previously published RNA-seq dataset (**Figure 6E**). This protein is most highly expressed in liver, skin, and brain tissue in healthy fish (**Figure 6F**). CFHL-1 was not correlated with bacterial load, nor was it correlated with STNC, Hapt, Cath, GDF-15, or Lept plasma levels (**Figure 5I**). However, it was negatively correlated with C1q-LP3 and CTNC. These data suggest that the rainbow trout *cfhl-1* gene produces both higher constitutive mRNA transcript and protein abundance in plasma in the resistant line and thus is a candidate biomarker of relative disease resistance.

### 3.8 Biomarker levels in commercial populations of rainbow trout and Atlantic salmon

**Figure 7:**
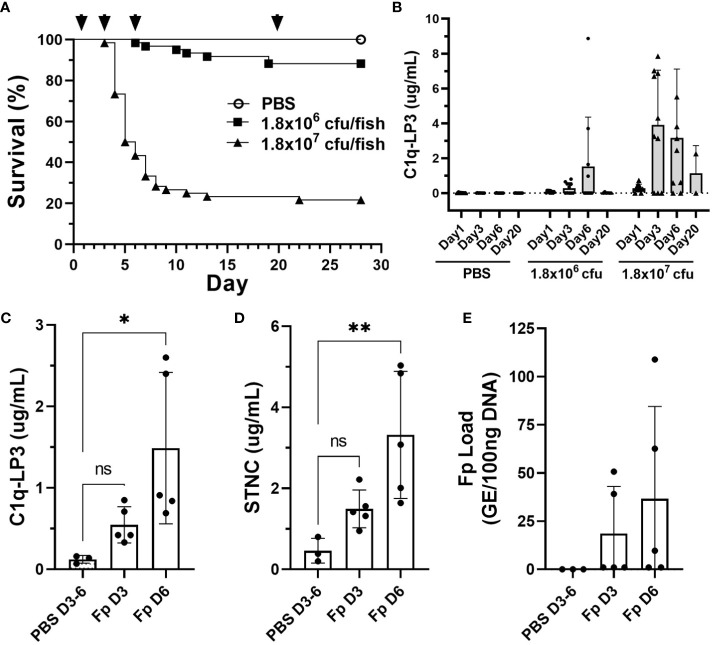
Biomarker elevation in commercial stocks of rainbow trout and Atlantic salmon challenged with *F. psychrophilum*. **(A)** Rainbow trout post-challenge survival; arrows indicate sampling time points D1, D3, D6, and D20 post-injection. **(B)** C1q-LP3 in plasma following challenge (mean ± sd). *N* = 10 fish per time point except for day 20 (*n* = 6 or 2; low and high dose, respectively). **(C)** Atlantic salmon C1q-LP3 at 3 and 6 days post-challenge, **(D)** STNC, and **(E)** *Fp* load in spleen. One-way ANOVA Kruskal–Wallis test with Dunn’s multiple comparisons test (mean and sd). Asterisks indicate significance values (* *p* < 0.05, ** *p* < 0.01).

To evaluate whether several of the biomarkers identified in this study might be informative in other rainbow trout stocks and salmonid species, we challenged a commercial line of rainbow trout and Atlantic salmon with *Fp* by i.m. injection. Rainbow trout survival following high dose, *Fp* CSF259-93 challenge (1.8 × 10^7^ cfu/fish) resulted in 78.3% mortality (*n* = 47/60), while the low-dose challenge (1.8 × 10^6^ cfu/fish) resulted in 11.7% mortality (*n* = 7/60) (**Figure 7A**). Elevated C1q-LP3 plasma levels were associated with higher challenge dose and peaked on days 3 and 6 (**Figure 7B**). In the low challenge dose, the C1q-LP3 plasma concentration peaked on day 6. Atlantic salmon possess a 1:1 orthologue of C1q-LP3 (**Supplementary Figure 6E**) and exhibited significantly elevated plasma levels on day 6 post-injection (**Figure 7C**) that was correlated with elevated STNC (**Figure 7D**) and elevated, but not significantly different, *Fp* load (**Figure 7E**).

## 4 Discussion

In this study, we investigate differences in plasma protein abundance between selectively bred rainbow trout lines to better understand the physiological differences associated with the resistant and susceptible phenotypes. At present, we do not know whether the increased survival of the resistant line is due to differences in constitutively expressed defenses or to differences in an inducible response following pathogen exposure, as has been described in other systems (62–64). We compared pools of plasma consisting of between 9 and 20 individuals from 10 families per line to minimize the interindividual variation, which can be considerable between fish (65, 66), and, thus, focus on the consistent differences associated with each genetic line. Sixty-seven infection-modulated proteins were identified exhibiting twofold differential abundance as well as a subset of constitutively expressed proteins that differed between lines. In agreement with prior RNA-seq data for these lines, we identified more infection-regulated proteins in the susceptible line (*n* = 61) compared to the resistant line (*n* = 35) at day 6 post-injection. The higher number in the susceptible line was associated with elevated bacterial spleen load and a lower blood packed cell volume. Using specific immunoassays, elevated STNC, haptoglobin, and Cath 2 were validated as infection-induced biomarkers in both lines. While C1q-LP3, leptin, GDF-15, and CNTC were robustly elevated in the susceptible line, they were weakly elevated in the infected resistant line, thus highlighting the differential physiological process occurring in each genetic line at this time point. Several proteins were constitutively differentially abundant, including CFHL-1 protein, which was higher in the resistant line.

### 4.1 Previous plasma and mucus proteomic studies in rainbow trout

Proteomic analyses of salmonid fish plasma and tissue provides a powerful approach to measure biological status (67), sea water adaption (68), and immunological response to defined antigens (65) and pathogens (69–74). The TMT 6-plex labeling in this study identified 513 plasma proteins and allowed comparison of relative abundance. The number of proteins is comparable to a study by Bakke et al. (65) who used label-free LC-MS and identified 600 rainbow trout plasma proteins, of which, 278 were consistently detected in all six study fish at seven time points. Most of the consistently detected proteins (~90%, 250/278) were also identified in this study, suggesting general agreement between label-free and isobaric approaches.

Hoare et al. (43) analyzed skin mucus proteome differences between *Fp* immersion-challenged and injection-challenged rainbow trout and identified two differentially expressed actin-related proteins. One of these, beta actin (NP_001117707.1), was also identified in our study, and while it did not meet the 2-fold criteria, it was upregulated ~1.5-fold in the SFp group compared to unhandled or PBS-injected fish.

### 4.2 Pathophysiology of infection and tissue damage

Our study demonstrated an elevation of intracellular proteins in plasma associated with muscle function. Furthermore, the corresponding genes were not transcriptionally regulated, suggesting that the proteins were being released from cells by leakage/cell death (75). This is consistent with observations of caveating muscle lesions along the dorsum of infected fish, which is a hallmark of BCWD (76). *Fp* secretes extracellular proteolytic enzymes that degrade collagen, fibrinogen, and hemoglobin (77, 78) as well as lytic factors for fish erythrocytes presumably to enhance nutrient acquisition. In the salmonid host, *Fp* is predicted to utilize proteinaceous compounds and fatty acids based on genomic pathway analyses and gene expression studies (79, 80). Iturriaga et al. (42) reported that 5-h coculture of *Fp* JIP02/86 with skeletal muscle cells from rainbow trout induced apoptosis at 48 hpi with caspase-3 and PARP-1 cleavage. Cytotoxicity required direct contact between bacteria and muscle cells and correlated with increased Iκbα and NF-κB protein repression. In our study, GO annotated terms for cytoskeleton, troponin complex, and myoglobinuria were overrepresented while protein levels of troponin I, fast skeletal muscle-like protein [XP_021454537] was four times higher in susceptible line fish than resistant line fish. Additionally, we confirmed by immunoassays that plasma STNC levels were significantly higher in infected fish and correlated with bacterial load supporting STNC as a biomarker for BCWD. Plasma CNTC levels were less conclusive, suggesting that cardiac muscle and/or slow-twitch skeletal muscle damage plays a reduced role compared to skeletal muscle damage as a sequela of *Fp* infection. This is consistent with myocarditis as an important (81) but less frequently observed histopathologic lesion in fish with BCWD (82).

Vertebrate tissue and blood are an iron-restricted environment with complex host–pathogen strategies for nutrient sequestration or acquisition (83). *Flavobacterium psychrophilum* produces two different heme/iron transport systems that are required for full virulence (84), emphasizing the importance of iron acquisition. In this study, the mean packed cell volume, a measure of anemia, fell below the established reference interval in the susceptible line fish (29) with multiple fish falling below the generally accepted critical level of 22% (85). The correlation between packed cell volume and bacterial load is consistent with previous studies (30) and indicates anemia as a confounding factor for survival. Infection was associated with upregulation of transcriptomic GO terms associated with normochromic and hemolytic anemia, and anemia due to reduced life span of red blood cells. Host heme-scavenging proteins were upregulated in both genetic lines during infection including haptoglobin, hepcidin, cathelicidin-1, and heme-binding protein 2-like protein. Hepcidin (chr2) has been identified as a candidate gene within a QTL interval associated with resistance to *Fp* immersion challenge (13). Recently, Ali and Salem (86) report the identification and differential expression of several long-noncoding natural antisense transcripts complementary to hemolysis-related genes on day 5 post-infection, in the same populations of fish used in this study, suggesting enhanced clearance of free hemoglobin and heme and possibly increased erythropoiesis. In summary, blood and muscle components are released into the plasma, enhancing bacterial growth in tissues and blood (40), and it is possible that these host components may also act as endogenous damage-associated molecular patterns driving NLRP-mediated inflammation (87, 88). Recently, Liu et al. (89) identified *nlrp1* on Chr8 as a candidate gene involved in genetic resistance. Future research exploring the pathophysiology of anemia and the divergence in severity between genetic lines may elucidate important mechanisms associated with survival.

Various anti-proteases were upregulated during infection with several being differentially regulated between genetic lines. Notably, alpha-2-antiplasmin-like protein [XP_021438281.1] was 4.0-fold higher in infected susceptible line fish and 2.7-fold higher in infected resistant line fish compared to PBS-challenged cohorts. Alpha-2-macroglobulin-like isoform X1 [XP_021437988.1] increased 2.7-fold and 1.7-fold in infected susceptible and resistant line fish, respectively, with a 1.8-fold higher abundance in the susceptible-line compared to the resistant line. Although anti-protease levels were not specifically examined for their correlation with pathogen load, elevated levels in susceptible line fish may be directly related to increased infection intensity and pathogen-derived proteolytic factors (90). Relatedly, anti-proteases may be upregulated in response to increased tissue injury and subsequent generation of inflammatory mediators (91). Differential responses between genetic lines may be secondary to higher severity of tissue inflammation and necrosis in susceptible line fish compared to resistant fish (82). Further proteomics studies are needed to clarify the origin and relevance associated with specific tissues.

### 4.3 A novel biomarker, plasma C1q-LP3, was elevated in the susceptible line after infection

The protein with the highest fold change between infected susceptible and resistant line fish was a C1q/TNFSF domain containing protein, designated C1q-LP3. Proteins belonging to the C1q and TNF superfamily in mammals are involved in diverse processes including inflammation, host defense, apoptosis, autoimmunity, cell differentiation, organogenesis, and insulin-resistant obesity (92, 93). C1q domain encoding genes are abundant in sequenced fish genomes with 52 genes reported in the zebrafish genome (94). In salmonids, 27 orthogroups containing a C1q pfam domain were identified that include a total of 38 different proteins (95). Comparison across genome-sequenced fish species suggests complex patterns of apparent contraction/loss and gene expansion (**Supplementary Figure 6D**). One-to-one orthologues of rainbow trout C1q-LP3 are present in pike (XP_010876306.1), *O. keta* (XP_035628802.1), and Atlantic salmon (XP_013983805.1) (**Supplementary Figure 6E** and (95)). Analysis of a commercial population of rainbow trout and Atlantic salmon challenged with *Fp* indicate increased plasma abundance, suggesting a conserved response across populations of trout and salmonid species. However, a direct 1:1 orthologue has not been identified in the genomes of *O. nerka*, *O. kisutch*, *O. tshawytscha*, and *O. gorbuscha*, and assuming these assemblies are correct, caution is thus warranted regarding the use of C1q-LP3 as a biomarker in these species.

The function of C1q-LP3 in salmonid fish is unknown. A search for the trout orthologue of C-reactive protein (CRP) identified a carbohydrate binding protein with a C1q-domain, designated C-polysaccharide-binding protein (TCBP1) (96–98). TCBP1 is 245 aa with a leader peptide and forms a trimer. C1q-LP3 is a similar size, 256 aa, and can trimerize in addition to forming higher-order oligomers (**Supplementary Figure 6A** and data not shown). Plasma levels of TCBP1 increased threefold 48 h after *Vibrio anguillarum* challenge with baseline values of 37 μg/mL and elevation to 117 μg/mL (96). This differs from C1q-LP3, which, in naive fish, has a lower baseline average of ~50 ng/mL that increased to a mean of 10.7 μg/mL in the ARS-Fp-S line on day 6 post-challenge (**Figure 5A**). The TCBP1 gene produces five different transcripts that are collectively increased several hundredfold in the liver with less expression in the anterior kidney and almost no expression in the spleen at 48 h after challenge with *Aeromonas salmonicida* (99). At present, we have not examined the tissue expression profile of the *c1qlp3* gene after challenge, but baseline expression levels in naïve fish were highest in the intestine, gill, and spleen with very little detected in the liver as measured by RNA-seq (**Supplementary Figure 6C**). TCBP1 transcript upregulation appears to be somewhat pathogen specific as challenge with *Yersinia ruckeri* failed to alter the hepatic transcript level in rainbow trout, even though the investigators utilized primers detecting all five mRNA isoforms (99, 100). TCBP1 overexpression in the HEK-293 cells increased the active form of NF-κB and resulted in cell death, suggesting a proapoptotic function (99). Recently, a *c1qtnf4* gene was upregulated 3 days post-infection in a clonal line of rainbow trout exhibiting high susceptibility to BCWD but was not regulated in two other lines with either intermediate or higher relative resistance (40). The protein encoded by the *c1qtnf4* gene, XP_021456913.1, was not identified in plasma in our study. Further research exploring the relationship between plasma C1q-LP3 levels and pathogen specificity, susceptibility, and tissue destruction/apoptosis will add to the understanding of role of C1q-domain containing proteins in teleost fish.

### 4.4 Acute phase response and additional plasma biomarkers of infection

Multiple acute phase response proteins were identified in the dataset including complement family and clotting components, lectins, and apolipoproteins (101, 102). We measured a representative acute phase response protein, haptoglobin, by specific ELISA and confirmed signification elevation in both the infected resistant and susceptible line fish (**Figure 6C**). Mean haptoglobin increased over 90-fold from 24 ng/mL to 2,374 ng/mL in the infected susceptible line and over 70-fold from 16 ng/mL to 1,160 ng/mL in resistant line fish. In mammals and some fish species, haptoglobin is recognized as a scavenger of free hemoglobin released from damaged erythrocytes; however, Redmond et al. (103) failed to isolate trout haptoglobin using immobilized trout hemoglobin calling into question whether this function is conserved in salmonids. In the Arlee rainbow trout genome assembly, there are four ohnologs and one pseudogene. Three genes are on chr6 (2 functional and one pseudogene), one gene is on chr 26, and one is located on an unplaced scaffold. Interestingly, we did not observe elevated plasma haptoglobin in Atlantic salmon challenged with *F. psychrophilum* using this same assay (data not shown). There are at least nine haptoglobin-like genes in the Atlantic salmon genome with most located on unplaced scaffolds, suggesting an interesting divergence between species. The functional role of elevated haptoglobin in the response to *Fp* infection remains to be determined.

The antimicrobial peptide cathelicidin 2 was identified as upregulated in both lines by proteomics and a specific ELISA (**Figure 6D**). Mean cathelicidin 2 increased over 20-fold from 1.2 μg/mL to 29.7 μg/mL in the infected susceptible line and over 10-fold from 1.0 μg/mL to 10.6 μg/mL in the resistant line. Cathelicidin is known to be upregulated in rainbow trout by bacterial infection as well as by IL-6 (104–106). To our knowledge, this is the first available immunoassay for this protein, and further evaluation as a general health biomarker is warranted.

Salmonid fish often display reduced appetite during Fp infection, and in our prior RNA-seq study (27), *gdf15* (Chr28) and *lep* (Chr2) gene transcripts were highly upregulated. In mammals, elevated plasma levels of GDF-15 or leptin have independent roles in appetite suppression and energy homeostasis and can modulate host response to pathogen infection (107, 108). In this study, we did not identify these proteins in our proteomics dataset presumably as the total abundance was too low for consistent detection. However, we developed two novel ELISAs for these proteins and investigated their utility as biomarkers of infection. Plasma GDF-15 increased over 50-fold from 3.5 ng/mL to 201.9 ng/mL in infected ARS-Fp-S line fish (**Figure 5F**). Similarly, plasma leptin levels increased over 20-fold from a baseline mean of 3.5 ng/mL to 79.4 ng/mL in infected ARS-Fp-S line fish (**Figure 5E**), indicating potential utility as infection biomarkers. Whether the observed infection-mediated appetite suppression is directly linked to elevated rainbow trout GDF-15 and/or leptin levels in this model requires further study. Both proteins can also have immunomodulatory roles. In mammals, increased circulating GDF-15 impairs the production of the proinflammatory/Type 1 response while enhancing the anti-inflammatory/Type 2 response (107). Elevated leptin can enhance the activity and function of granulocytes, monocytes, macrophages, natural killer cells, and T cells (109, 110). Leptin can also stimulate the production of proinflammatory cytokines, including IL-1β, IL-6, and TNF-α. Further research is required to determine whether leptin and GDF-15 modulate the differential response between lines and their utility as biomarkers of altered physiology in farmed fish.

### 4.5 CFHL-1 is an elevated biomarker associated with disease resistance

The plasma protein with the greatest constitutive differential abundance between genetic lines was a protein we designated CFHL-1 (LOC110524788). CFHL-1 is located on Chr5 adjacent to trout CFH (LOC100136111) and contains five short consensus repeats (SCP), also known as sushi domains (**Figure 5A**). Anastasiou et al. (111) first reported the cloning of trout complement factor H; however, the reported protein (401 aa) appears most similar (95%ID, 381/401 aa) to the C-terminus of the 705-aa protein encoded by LOC110524789 (XP_036834975.1), which is related to, but not the currently identified, trout orthologue of mammalian CFH. In humans, there are five Complement Factor H related (CFHR) proteins that can form homo- as well as heterodimers that bind to complement component C3b, and these genes are located in one locus on Chr1 (112). In humans, the entire chromosomal segment with the CFHR genes contains several large genomic repeat regions that allow non-homologous recombination and result in rearrangements with diverse outcomes including deletions and duplications (112). Interestingly, genetic polymorphisms in this region are associated with susceptibility to several bacterial diseases (113, 114) and CFH and CFHR proteins are targets for pathogen modulation (115). It should be noted that the rainbow trout gene models considerably differ between NCBI and Ensembl automated annotation. Additional long-read sequencing is required for the validation of these gene models. To date, we are unaware of any reports of variation in expression of trout CFH and related genes. A comprehensive comparison of 36 complement components in two rainbow trout strains (BORN and Troutlodge) identified similar expression for most genes including CFH but did not measure the additional CFHL genes (116).

This study identified two additional plasma proteins that were >2-fold higher in the resistant line compared to the susceptible line including trace amine-associated receptor 8a-like (XP_021435707.1) and ladder lectin-like (XP_021452379.1). There are 23 ladder lectin-like genes present in the Arlee genome but one matching LOC110519767 (from Swanson reference) was not identified. Further effort is warranted to determine whether any of these proteins that are elevated in the resistant line have a direct antimicrobial role.

### 4.6 Study limitations

There are several limitations within this study. First, we utilized a high-dose intramuscular injection challenge to infect large rainbow trout and aspects of the response may differ if fish are challenged by immersion (13), cohabitation, or natural challenge on-farm (12). Thus, the biomarkers associated with susceptibility and resistance phenotypes described here require further validation. Second, the version of the reference genome impacted the identification of some of the differentially abundant proteins. Genomes often contain regions of incomplete coverage, misassembly, and/or collapse. Both the C1q-LP3 and CFHL1 gene/protein models in NCBI changed between the Omyk1.0 and OmykA1.1 versions (**Supplementary Figure 5**), although the relative fold expression was consistent. However, for other genes/predicted proteins such as fibrinogen alpha chain-like (XP_021477882.1) and intelectin-like (XP_021452379.1), the proteins were either not present or fold change between groups/lines was not reproduced. Furthermore, gene models often differ between NCBI (used here) and Ensembl, especially for tandem, sequence-related genes. The availability of additional genome sequences combined with long-read transcript sequencing, and ultimately a pangenome reference, will facilitate accurate protein identification and abundance comparison. It is likely that reanalysis of the available dataset using an improved reference will uncover additional regulated proteins. Third, salmonid fish have multiple copies of some genes and resolving paralogues based on minor nt/aa differences is challenging. For example, the resolution of cathelicidin, haptoglobin, hepcidin, heme-binding protein 2, intelectin-like, and ladder lectin-like paralogues will require further effort to precisely distinguish between the tandem variants. Finally, here we quantified the plasma proteins with highest abundance, but many more proteins are present in plasma that are below detection with the methods used here (75). Other strategies such as albumin depletion (117) or specific immunoassays are required to detect and quantify proteins below the ~100 ng per mL range.

### 4.7 Conclusions

The plasma proteomes of susceptible and resistant line rainbow trout reflect immunologic and physiologic processes during *Fp* infection with differential responses that could be attributed to genetic line and divergent survival. Consistent with prior studies, bacterial load was not controlled in most susceptible line fish and tissue damage, inflammation, and acute phase response were highly elevated by day 6 post-challenge, which is a time point when differential mortality is observed. Elevated markers of tissue damage included STNC and, to a lesser extent, CTNC, while haptoglobin, cathelicidin, and PCV correlated with bacterial load. Plasma levels of these proteins may provide means to monitor disease progression in laboratory and aquaculture settings as biomarkers of infection (118). Proteins associated with genetic line included a novel susceptibility biomarker, C1q-LP3, and CFHL-1, which was identified as a baseline differentiator in resistant line fish. Although the premise that plasma biomarkers provide an accurate prediction for *Fp* resistance remains to be fully validated, TMT analysis coupled with genomic and transcriptomic characterization, and validation of protein levels using immunoassays provided a robust characterization of this model of disease resistance. Future studies that follow the time course of biomarker abundance and load in live fish will be useful for mapping the physiological trajectories that distinguish disease and recovery (119). Also, assessment of extrinsic factors that may affect plasma protein production and degradation such as diet, metabolic shifts, and pathogen virulence factors is warranted. Further elucidation of these processes will aid in the development of biomarker reference intervals for evaluating resistant line fish and health monitoring in aquaculture systems.

## Data availability statement

The data presented in the study are deposited in the ProteomeXchange Consortium repository, accession number PXD041308. All other data is included in Supplementary Data Files.

## Ethics statement

The animal study was approved by Fish were maintained at the NCCCWA and animal procedures were performed under the guidelines of IACUC Protocols #053, #076, #106 and #189. The study was conducted in accordance with the local legislation and institutional requirements.

## Author contributions

GW: Conceptualization, Data curation, Formal Analysis, Funding acquisition, Investigation, Project administration, Supervision, Visualization, Writing – original draft. DM: Conceptualization, Formal Analysis, Investigation, Writing – original draft. CC: Conceptualization, Data curation, Formal Analysis, Investigation, Methodology, Writing – review & editing. KO: Data curation, Methodology, Writing – review & editing. RR: Methodology, Writing – review & editing. TL: Data curation, Formal Analysis, Resources, Writing – review & editing.

## Supplementary material

Supplementary Figure 1 SDS-PAGE comparison of pooled samples and protein concentration standardization prior to TMT-Proteomic analyses. Nanodrop= protein concentration quantified by A_280_. Gel con = protein concentration quantified by SDS-PAGE. The correlation between assays was 0.69.

Supplementary Figure 2
**(A)** Bacterial load by genetic line and sorted by family geometric mean. Dotted line indicates genometric mean for each genetic line. **(B)** Mean family survival challenged at ~80 days post-hatch. **(C)** Predicted family breeding value using the entire phenotypic and pedigree dataset. ARS-Fp-R line families, blue circles; ARS-Fp-S line families, red squares. **(D)** Packed cell volume (mean and sd) and 90 percent reference interval. Mortalities are indicated by an * and are not included in the PCV figure.

Supplementary Figure 3 Comparison of protein and mRNA abundance data by genetic line and infection status. **(A)** S-line PBS injected; **(B)** R-line PBS injected; **(C)** S-line Fp injected; **(D)** R-line Fp injected. Data are *Log*_2_ transformed day 5 post-challenge transcript abundance (TPM, Marancik et al., 2015) and day 6 post-challenge raw protein abundance values (**Supplemetary Data 2**). Both datasets used the Omyk_1.0 assembly (Swanson clonal line) as reference. Linear regression line and 95% CI, each slope is significantly non-zero.

Supplementary Figure 4 GO enrichment analysis of 2 fc regulated SFp proteins.

Supplementary Figure 5
**(A)** Alignment of C1q-LP3 and **(B)** Cfhl1 proteins predicted from rainbow trout Omyk 1.0 (XP_021445926.1, XP_021460375.1) and USDA_OmykA_1.1 (XP_036824181.1, XP_036834653.1) genome assemblies respectively.

Supplementary Figure 6 C1q-LP3 protein structure, expression and orthologues. **(A)** SMART domain prediction and ribbon/space filling models of the C1Q/TNFSF domain. **(B)** Immunoprecipitation and western blot using affinity purified rabbit anti-C1q-LP3: lanes 1) recombinant his-tagged C1q-LP3, 2) SFp pooled plasma, 3) RFp pooled plasma. **(C)** Transcript counts in normal tissue from the Swanson clonal line. **(D)** Ensembl gene gain/loss tree prediction of ENSOMYG00000056356 (LOC110509328) based on the USDA_OmykA_1.1 genome assembly (Arlee clonal line). **(D)** 1:1 predicted orthologues in Atlantic salmon, brown trout and Huchen (Ensembl release 108).

Supplementary Figure 7 Development of assays to measure C1q-LP3 in fish plasma or serum. **(A)** Recombinant C1q-LP3 protein produced in E. coli used to generate affinity purified rabbit antibody. **(B)** Capture ELISA standard curve and limit of detection. **(C)** Comparison of SPARCL and ELISA assays. **(D)** Linearity of serial dilutions of either denatured or native plasma samples having high concentrations of C1q-LP3.

Supplementary Figure 8 Plasma CNTC measured by SPARCL assay. Asterisk indicates significance value (* *P*<0.05).

Supplementary Figure 9 Heat map of markers significantly associated with infection (q<0.05) in the susceptible line **(A)** and resistant line **(B)**. Nearest network analysis of variables and projection in top three principal component space for the susceptible line **(C)** and resistant line **(D)**.

Supplementary Data Sheet 1 Fish ID, genetic line, family, infection status, load and biomarker data.

Supplementary Data Sheet 2 Proteomic analysis including abundance ratios, peptides and analysis parameters using either the Omyk_1.0 or OmykA_1.1 genome assemblies as reference.

Supplementary Data Sheet 3 Cross-reference of proteomic, RNA-Seq and normal tissue expression datasets.
