# Supplementary figures and images for: Plasma proteomic profiling of bacterial cold water disease-resistant and -susceptible rainbow trout lines and biomarker discovery
Source: Front Immunol. 2023 Oct 20;14:1265386. doi: 10.3389/fimmu.2023.1265386 (PMC10623068; doi:10.3389/fimmu.2023.1265386)

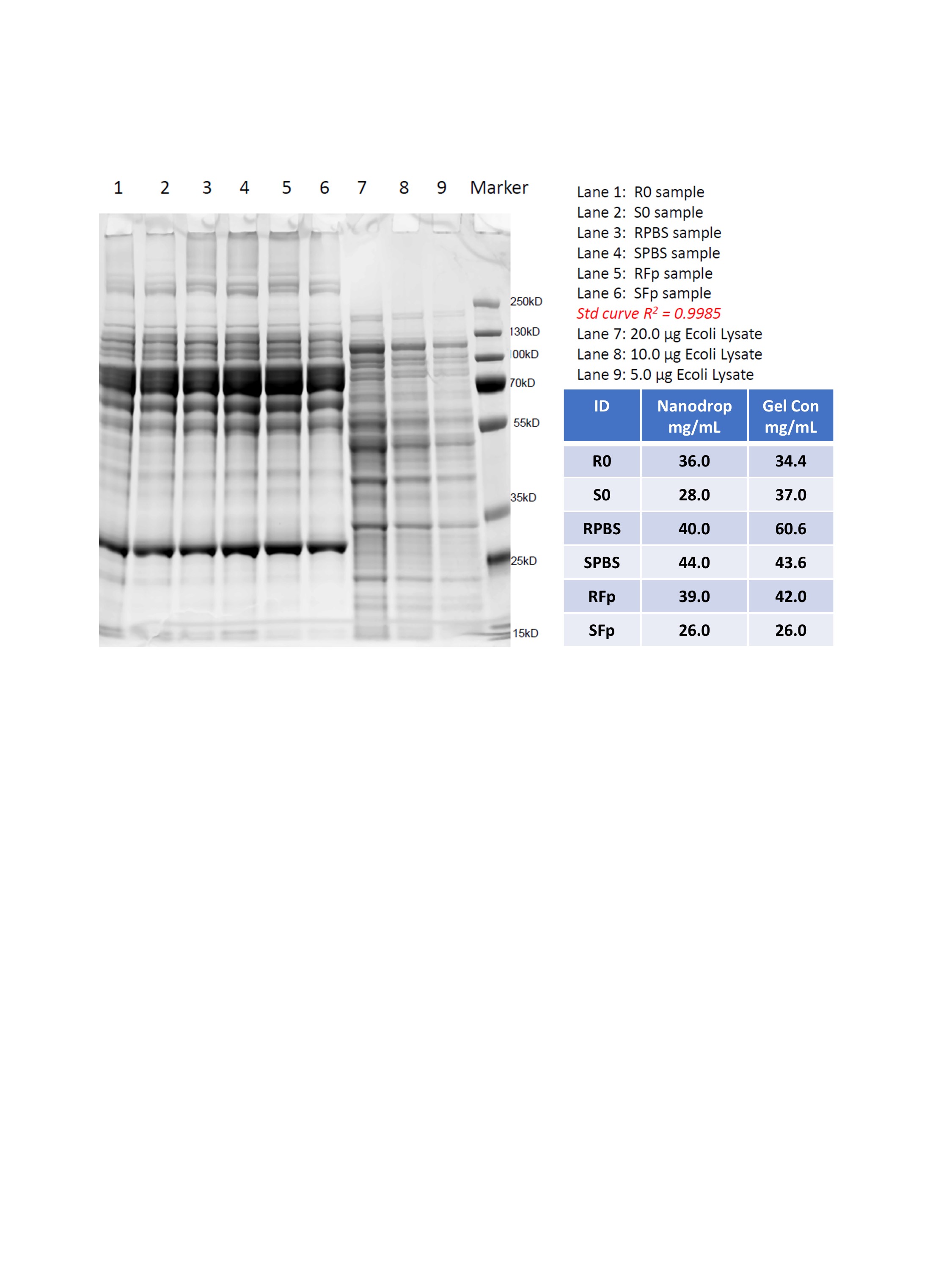

Supplement: Supplementary Figure 1 — SDS-PAGE comparison of pooled samples and protein concentration standardization prior to TMT-Proteomic analyses. Nanodrop= protein concentration quantified by A280. Gel con = protein concentration quantified by SDS-PAGE. The correlation between assays was 0.69. [file Image_1.jpg]

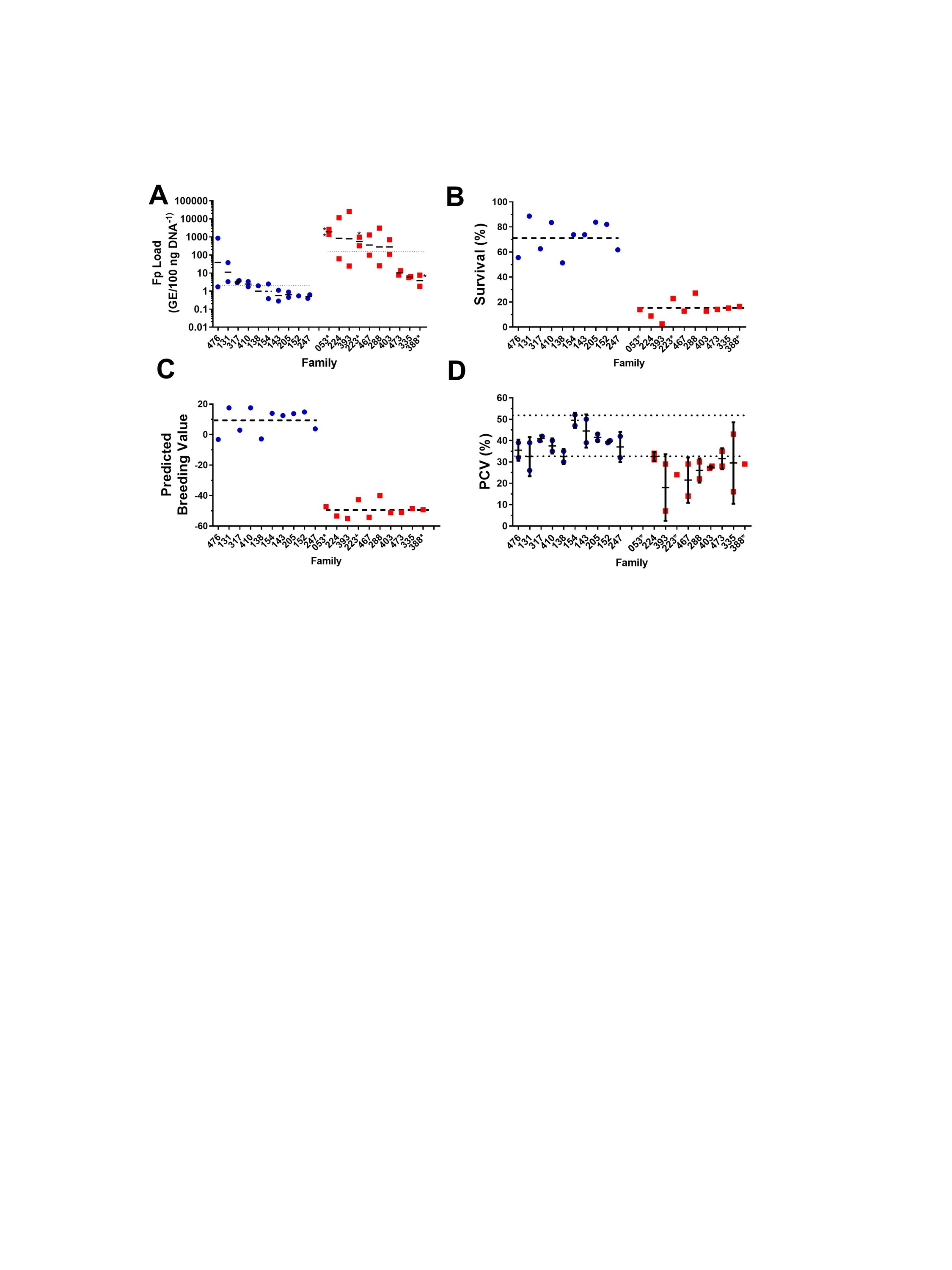

Supplement: Supplementary Figure 2 — (A) Bacterial load by genetic line and sorted by family geometric mean. Dotted line indicates genometric mean for each genetic line. (B) Mean family survival challenged at ~80 days post-hatch. (C) Predicted family breeding value using the entire phenotypic and pedigree dataset. ARS-Fp-R line families, blue circles; ARS-Fp-S line families, red squares. (D) Packed cell volume (mean and sd) and 90 percent reference interval. Mortalities are indicated by an * and are not included in the PCV figure. [file Image_2.jpg]

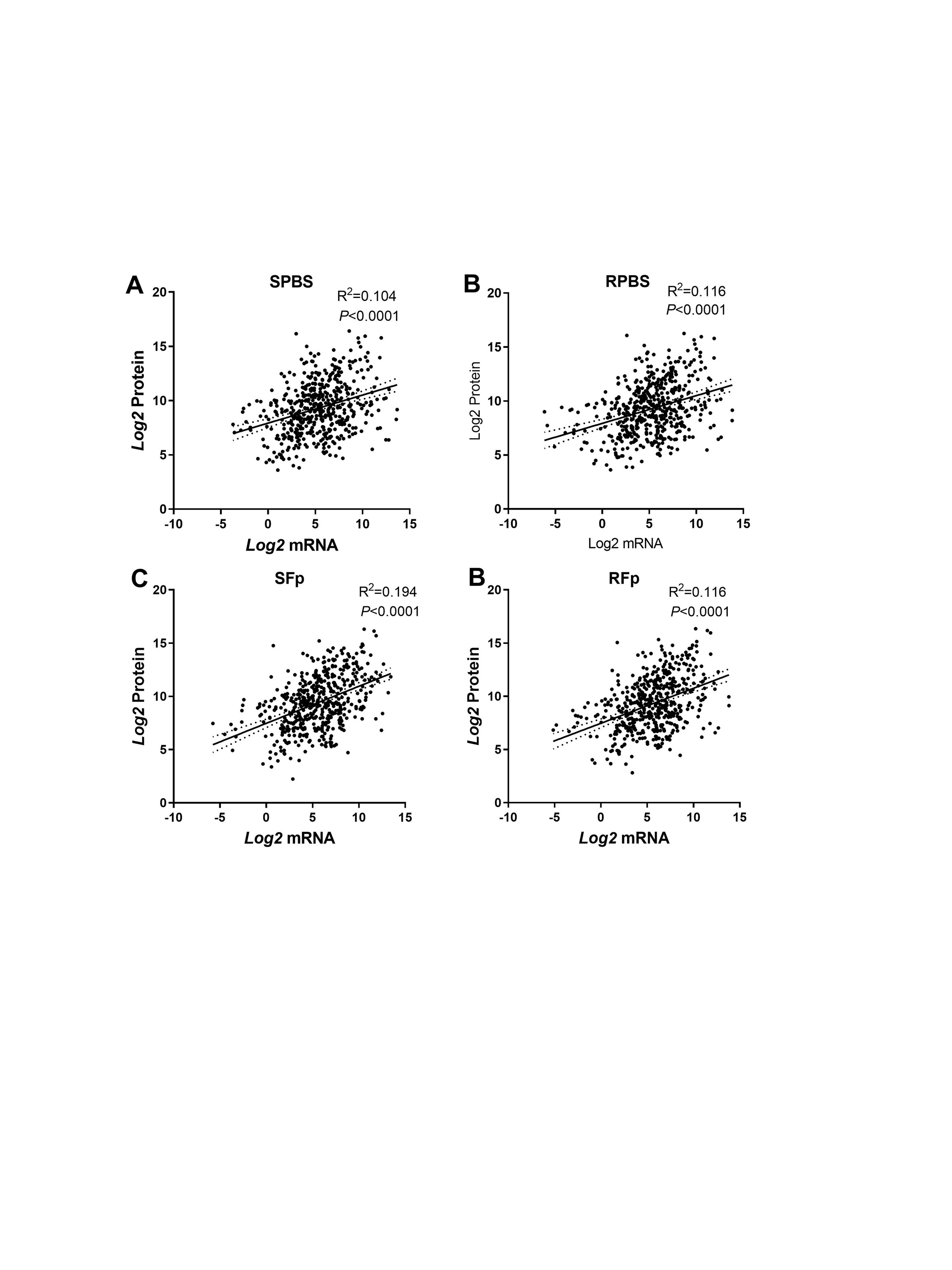

Supplement: Supplementary Figure 3 — Comparison of protein and mRNA abundance data by genetic line and infection status. (A) S-line PBS injected; (B) R-line PBS injected; (C) S-line Fp injected; (D) R-line Fp injected. Data are Log 2 transformed day 5 post-challenge transcript abundance (TPM, Marancik et al., 2015) and day 6 post-challenge raw protein abundance values ( Supplemetary Data 2 ). Both datasets used the Omyk_1.0 assembly (Swanson clonal line) as reference. Linear regression line and 95% CI, each slope is significantly non-zero. [file Image_3.jpg]

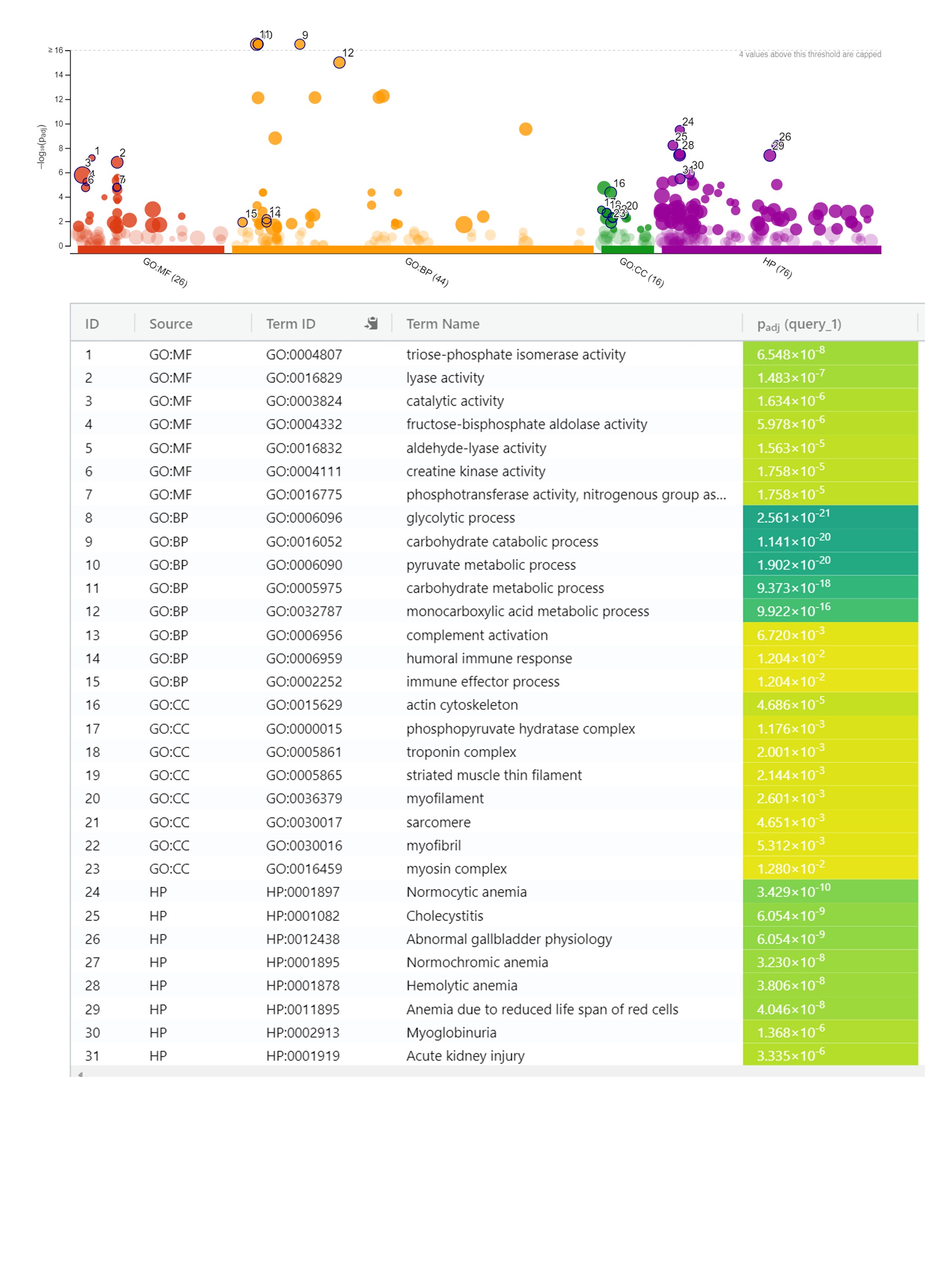

Supplement: Supplementary Figure 4 — GO enrichment analysis of 2 fc regulated SFp proteins. [file Image_4.jpg]

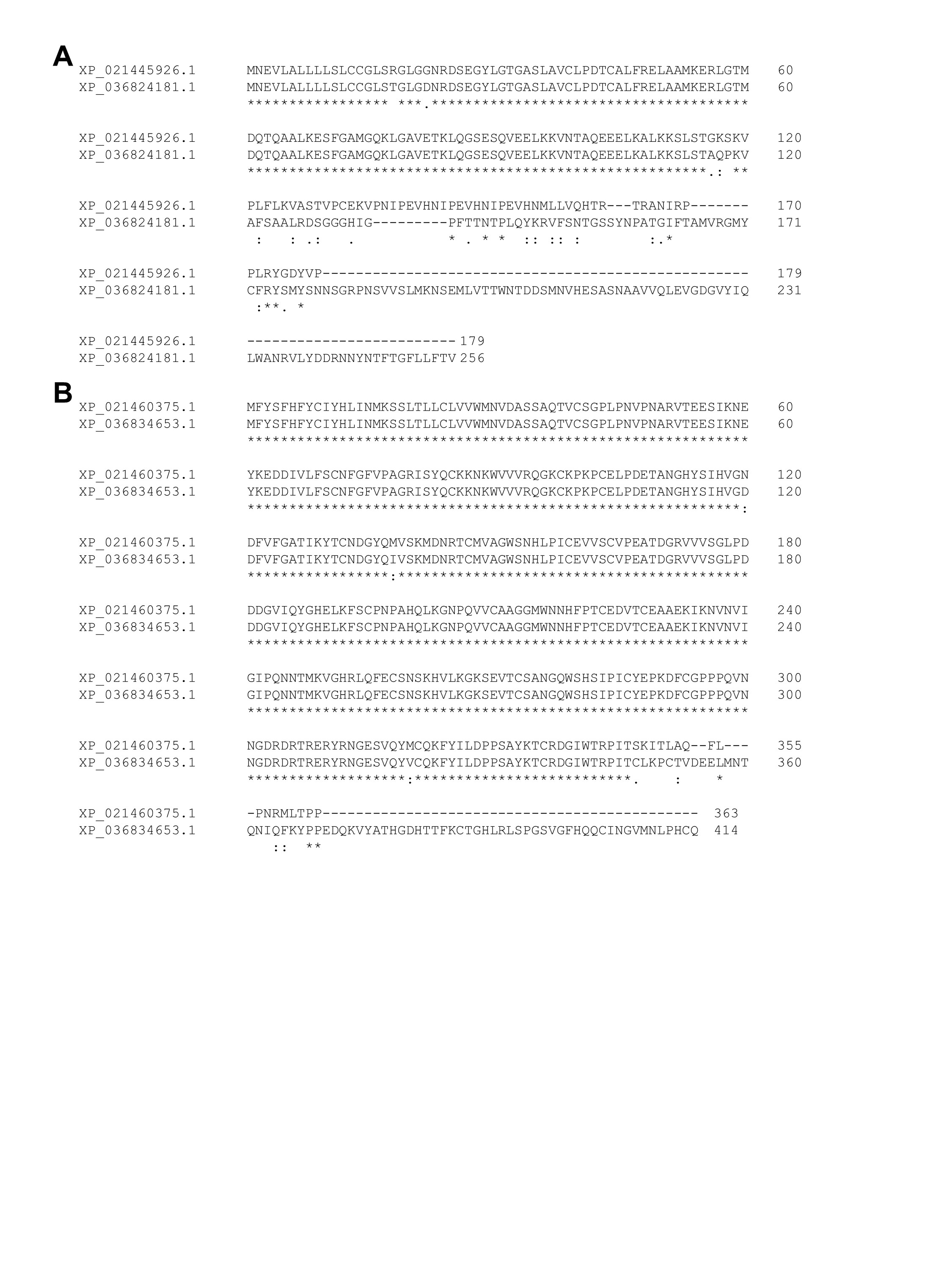

Supplement: Supplementary Figure 5 — (A) Alignment of C1q-LP3 and (B) Cfhl1 proteins predicted from rainbow trout Omyk 1.0 (XP_021445926.1, XP_021460375.1) and USDA_OmykA_1.1 (XP_036824181.1, XP_036834653.1) genome assemblies respectively. [file Image_5.jpg]

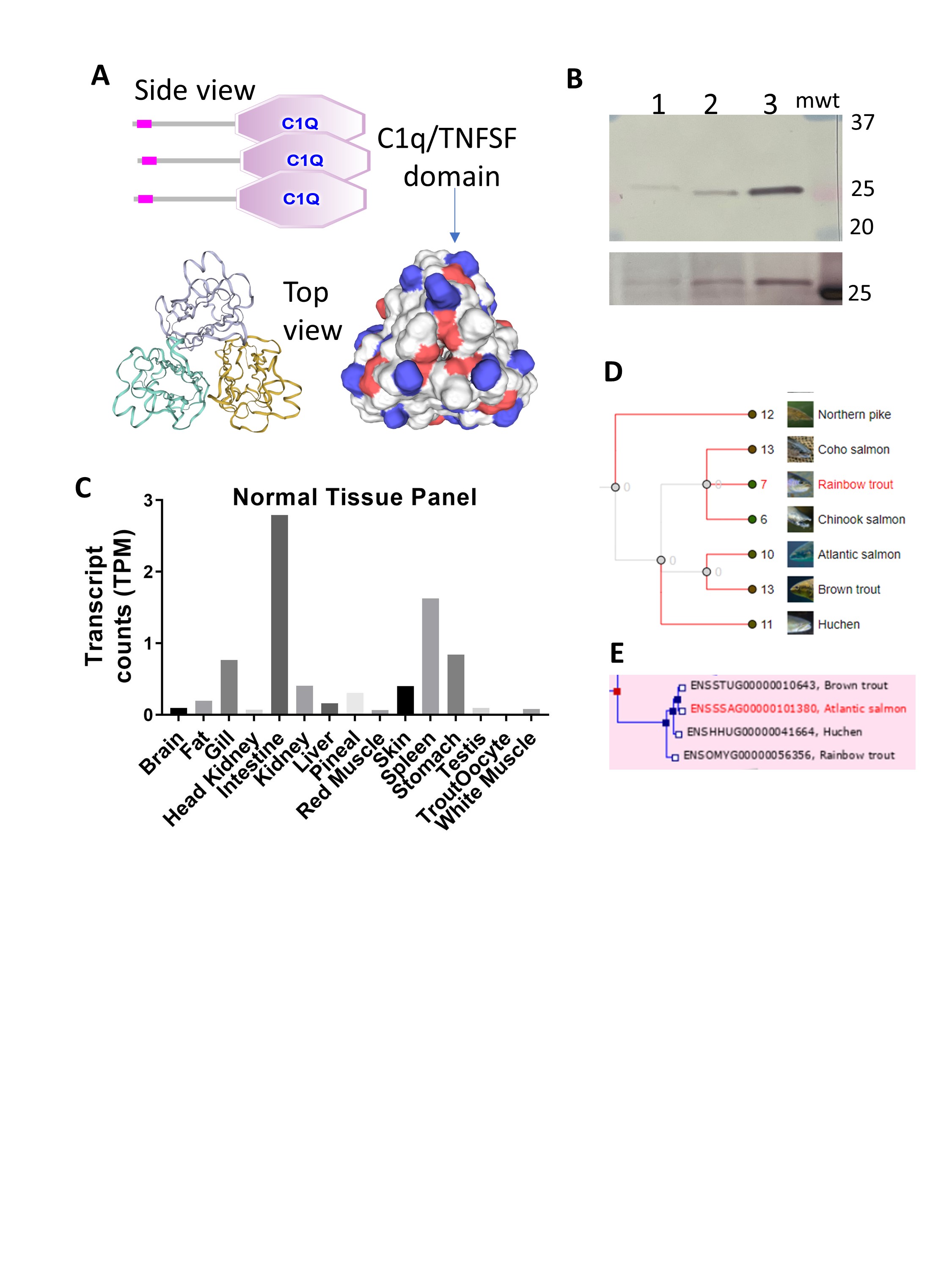

Supplement: Supplementary Figure 6 — C1q-LP3 protein structure, expression and orthologues. (A) SMART domain prediction and ribbon/space filling models of the C1Q/TNFSF domain. (B) Immunoprecipitation and western blot using affinity purified rabbit anti-C1q-LP3: lanes 1) recombinant his-tagged C1q-LP3, 2) SFp pooled plasma, 3) RFp pooled plasma. (C) Transcript counts in normal tissue from the Swanson clonal line. (D) Ensembl gene gain/loss tree prediction of ENSOMYG00000056356 (LOC110509328) based on the USDA_OmykA_1.1 genome assembly (Arlee clonal line). (D) 1:1 predicted orthologues in Atlantic salmon, brown trout and Huchen (Ensembl release 108). [file Image_6.jpg]

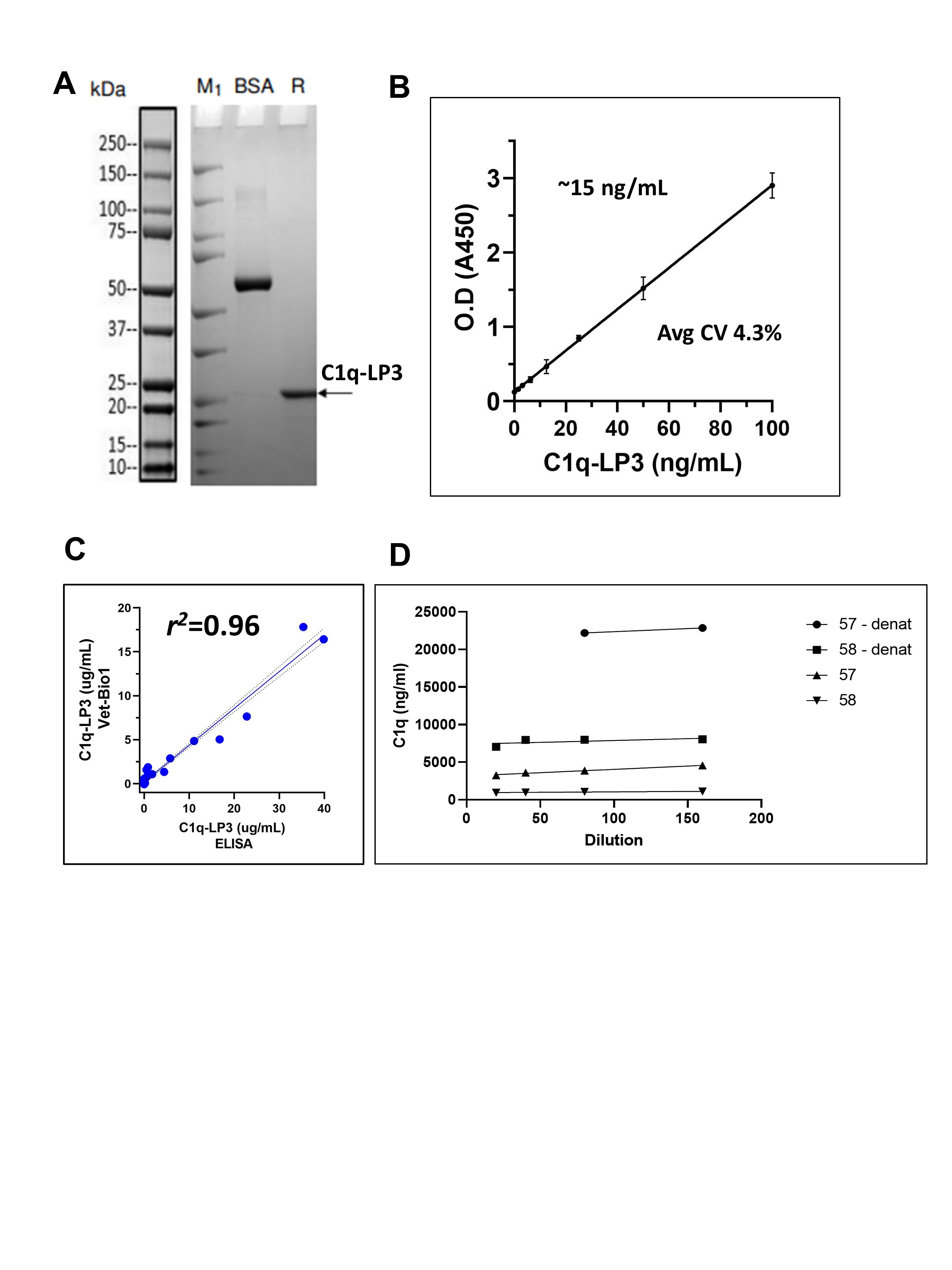

Supplement: Supplementary Figure 7 — Development of assays to measure C1q-LP3 in fish plasma or serum. (A) Recombinant C1q-LP3 protein produced in E. coli used to generate affinity purified rabbit antibody. (B) Capture ELISA standard curve and limit of detection. (C) Comparison of SPARCL and ELISA assays. (D) Linearity of serial dilutions of either denatured or native plasma samples having high concentrations of C1q-LP3. [file Image_7.jpg]

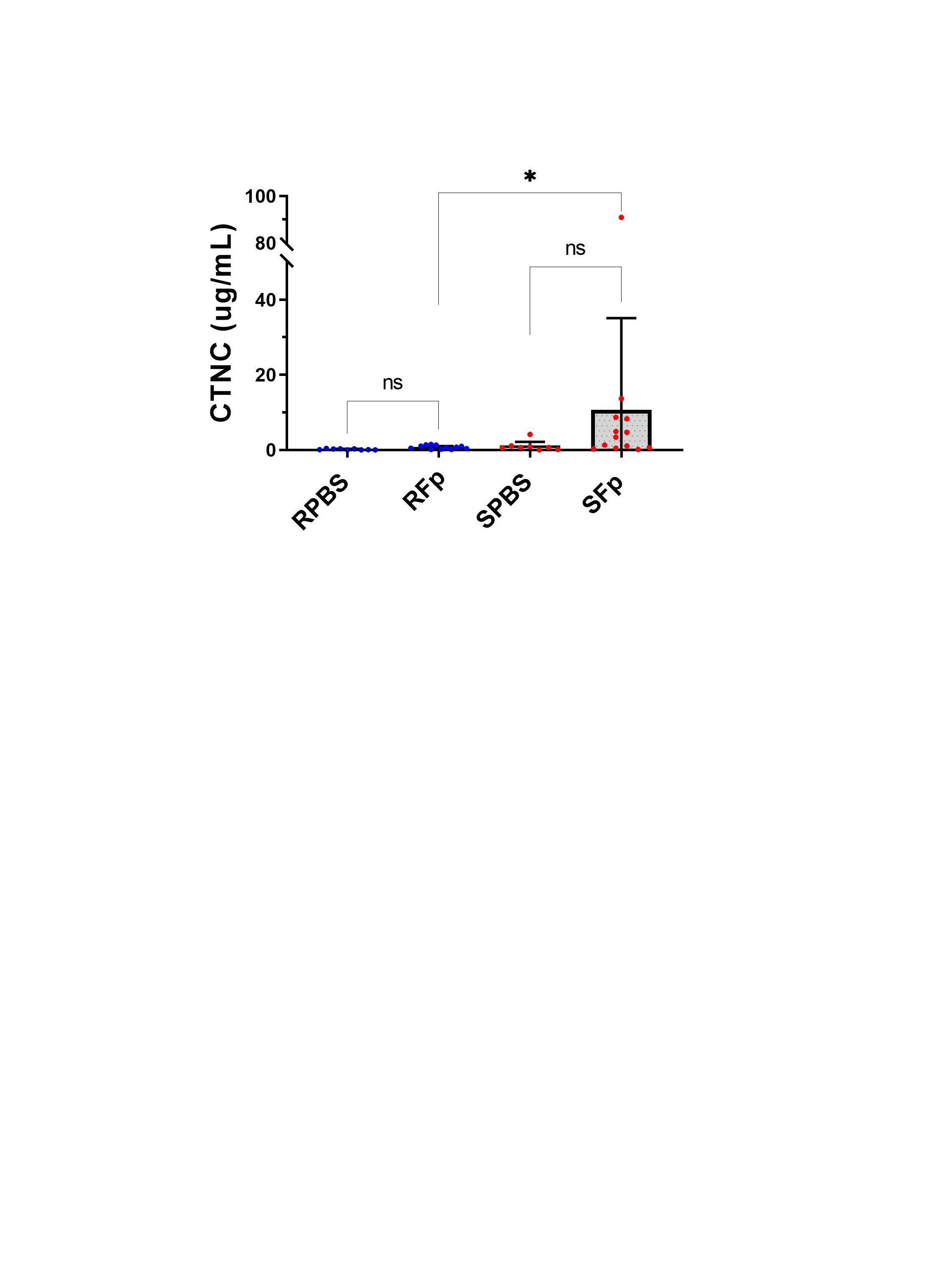

Supplement: Supplementary Figure 8 — Plasma CNTC measured by SPARCL assay. Asterisk indicates significance value (* P<0.05). [file Image_8.jpg]

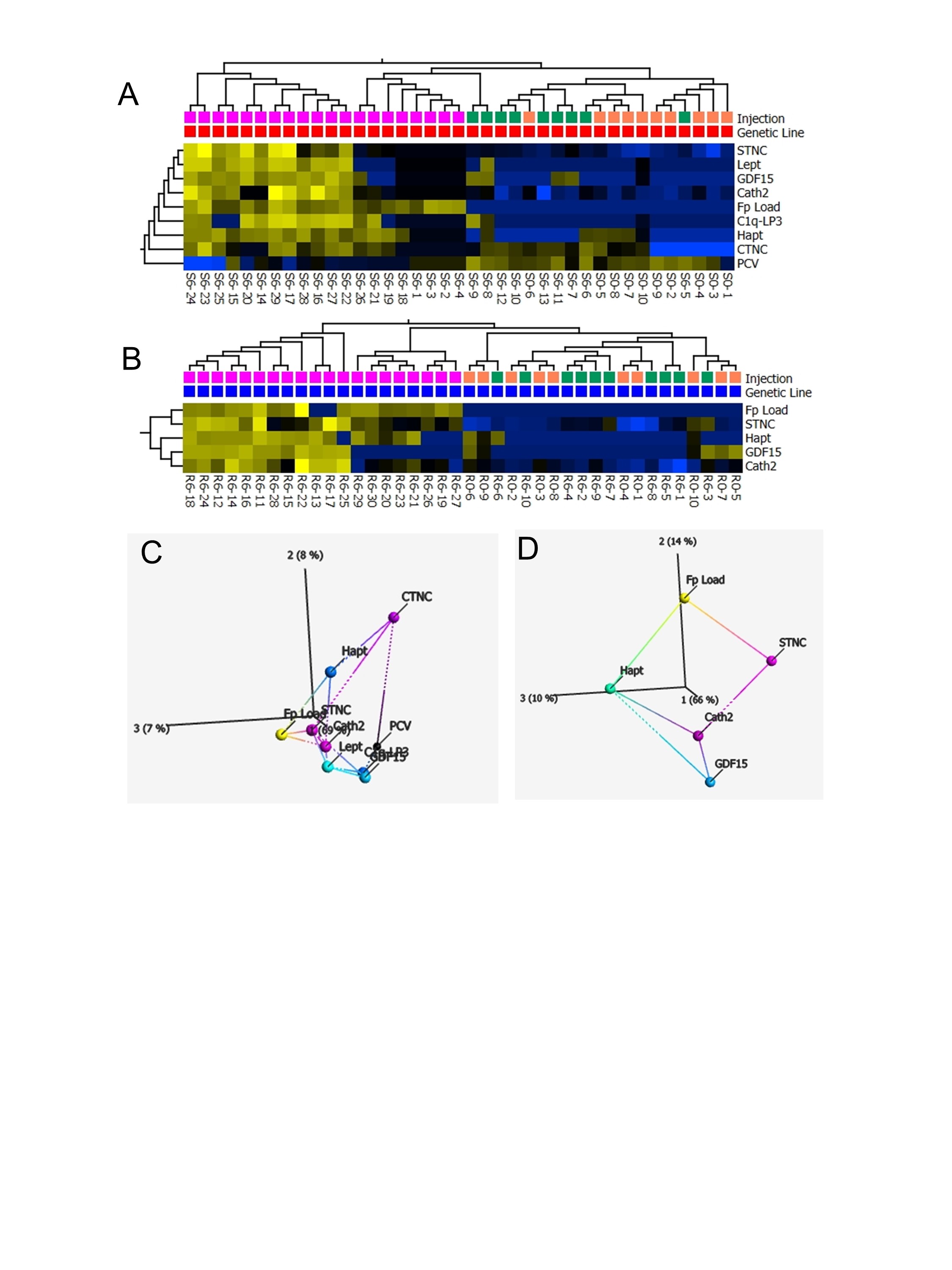

Supplement: Supplementary Figure 9 — Heat map of markers significantly associated with infection (q<0.05) in the susceptible line (A) and resistant line (B). Nearest network analysis of variables and projection in top three principal component space for the susceptible line (C) and resistant line (D). [file Image_9.jpg]
